# Synergistic mediating effect of edible fungal polysaccharides (*Auricularia* and *Tremellan*) and *Crataegus* flavonoids in hyperlipidemic rats

**DOI:** 10.1002/fsn3.3459

**Published:** 2023-06-15

**Authors:** Ke Shi, Tao Zhou, Yu‐fei Yuan, Dan‐dan Li, Bin‐bin Gong, Shan Gao, Qi‐jia Chen, Yan‐dong Li, Xue Han

**Affiliations:** ^1^ College of Food Science and Biology Hebei University of Science and Technology Shijiazhuang China; ^2^ College of Biological Science and Engineering Xingtai University Xingtai China; ^3^ Hebei Provincial Station of Veterinary Drug and Feed Shijiazhuang China

**Keywords:** flavonoids, hyperlipidemia, intestinal microflora, polysaccharides, synergistic

## Abstract

Both edible fungal polysaccharides (*Auricularia* and *Tremellan*) and *Crataegus* flavonoids promote the balance of dyslipidemia, which have a positive biological regulating effect on intestinal flora. In this study, the extraction of water‐soluble polysaccharides from *Auricularia* and *Tremellan* was investigated and optimized firstly. Polysaccharides and flavonoids were then combined to study the effects on the mediating role of abnormal blood lipid concentration and intestinal flora in vivo. The rats were divided into 10 groups, the NC (normal control), HM (model), PCI (Simvastatin control), PCII (Fenofibrate control), AAP (*Auricularia auricular* Polysaccharide), TFP (*Tremella fuciformis* Polysaccharide), HF (*Crataegus* Flavonoid), LDC (Low‐dose combination), MDC (Medium dose combination), and HDC (High‐dose combination), used to explore the impact of polysaccharides and flavonoids complex on state of blood lipid, liver, and intestinal flora of dyslipidemia rats. The results showed that the combination of polysaccharides and flavonoids could significantly decrease the levels of triglyceride (TG), total cholesterol (TC), low‐density lipoprotein (LDL‐C), and increase the level of high‐density lipoprotein cholesterol (HDL‐C). It also significantly decreased the levels of alanine aminotransferase (ALT) and aspartate aminotransferase (AST) and improved liver morphology. What is more, the HDC favorably alters the intestinal microflora balance, promotes intestinal integrity and mobility, and inhibits the growth of harmful bacteria such as *Escherichia coli/Shigella* and *Clostridium* compared with HM group. In brief, the combination of polysaccharides and flavonoids had a synergistic effect on the remission of dyslipidemia, and promoted health by improving lipid metabolism, protecting liver tissue, and regulating the intestinal flora in hyperlipidemia rats.

## Graphical abstract

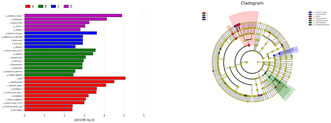

## 1 INTRODUCTION

Hyperlipemia (HLP) usually refers to abnormalities in lipid metabolism and induces cardiovascular diseases such as hypertension, coronary heart disease, and atherosclerosis. Hyperlipidemia is a condition of abnormal lipid metabolism in the body which is due to environmental, genetic, and other factors or the factors' interaction. Hypolipidemic drugs are mainly used to control blood lipids within a normal range for a long time, and cannot completely cure hyperlipidemia. Clinically, statins, clobetin, nicotinic acid, and bile acid integrated resins are often used to prevent and treat hyperlipidemia with good results, but they have side effects inevitably (Hu & Ji, 2022; Pilotto et al., 2016). Because of the single target of chemical synthetic drugs, the effect on complex hyperlipidemia is poor (Ma, 2006). Traditional Chinese medicine is capable of multitarget action and multicomponent synergy, which has attracted much attention on new lipid‐reducing drugs' research (Liu, Liu, et al., 2022; Liu, Yanneng, et al., 2022).

Some polysaccharides can reduce blood lipids, especially fungal polysaccharides. Fungal polysaccharides come mainly from the Basidiomycetes family (and some from the Ascomycetes) and medicinal mushrooms have been widely used in Asia as part of traditional diet and medicine, and the utilization of their medicinal properties in naturally produced pharmaceuticals (Giavasis, 2014; Sun et al., 2022). Research has shown that *Tremella* and *Auricularia* polysaccharide can improve hyperlipidemia (Yang et al., 2019). Besides, fungal polysaccharides can regulate the movement of intestinal flora, improve the intestinal microenvironment, and can secrete health factors; play an important role in human nutrition absorption and metabolism (Conlon & Bird, 2014; Tong et al., 2023). Related experiments show that soluble polysaccharides can selectively promote the growth and reproduction of one or more intestinal flora, and produce health factors beneficial to the host.

*Crataegus* has been regarded as a superior supplement of “medicine and food homology”. *Crataegus* fruit has been approved by the Ministry of Health of the People's Republic of China as a functional food raw material and has been included as Chinese herbal medicine by the Chinese Pharmacopoeia (T. Li et al., 2013). *Crataegus* contains a variety of biologically active substances, in which polyphenols are one of the most important and effective ingredients, and their pharmacological effects are mainly due to flavonoids. Flavonoids are mostly crystalline solids, and a few are amorphous powders. They are hardly soluble in water and easily soluble in organic solvents. Some people (Feng et al., 2011) studied the blood lipids and antioxidant effects of perilla leaf chemical components and total flavonoids. At doses of 50–200 mg/kg, oral administration of TFP to hyperlipidemia rats was highly effective in decreasing the levels of serum total cholesterol (TC), triacylglycerols (TG), low‐density lipoprotein‐cholesterol (LDL‐C), and adipose tissue lipid accumulation, increasing the levels of serum high‐density lipoprotein‐cholesterol (HDL‐C), adjusting metabolic disturbance of lipoprotein, increasing antioxidant enzyme activity, and repressing development of atherosclerosis.

Therefore, we hypothesized combination of fungal polysaccharides and flavonoids could reduce blood lipids and positively regulate intestinal flora. To test the hypotheses, we combined the three components of *Auricularia auricula* polysaccharides (AAP), *Tremella fuciformis* polysaccharides (TFP), and *Crataegus* flavonoids (HF) to probe their effects on the levels of blood lipids and the regulation of the intestinal flora.

## 2 MATERIALS AND METHODS

### 2.1 Materials

*Auricularia auricula* and *tremella* used in the experiment were provided by Zhongkang Vegetable Planting Co., Ltd. The liquid nitrogen freezing pulverization method is used to pretreat the raw materials. The *Crataegus* flavonoids were provided by the Nutrition and Functional Food Laboratory of Hebei University of Science and Technology.

High‐fat diet formula: 78.8% of basic feed, 10% of lard, 10% of yolk powder, 1% of cholesterol, and 0.2% of cholate; normal diet, provided by Hebei Medical University.

### 2.2 Animals and groups

SPF Wistar rats (weighing 200 ± 20 g) were purchased from the Experimental Animal Center of Hebei Medical University (SCXK (JI) 2018‐004, 1410014, Shijiazhuang, China). All experiments were conducted following the Guide for the Care and Use of Laboratory Animals of National Institutes of Health. The animals were maintained in an environment with controlled temperature (20–25°C) and humidity (50 ± 5%), with food and water available at any time and natural light. The health of the rats was monitored every day. Before blood collection, rats were anesthetized with ether, and the other rats were euthanized by cervical dislocation under anesthesia with isoflurane.

The 90 rats were divided into 10 groups randomly, and the rats were given preventive treatment. According to previous studies (Bai et al., 2022), the ratio of recombination was determined to be AAP:TFP:HF = 1:1:1. The specific treatment methods are shown in Table 1. The experimental dose and feed intake are designed by the daily intake of flavonoids and polysaccharides. The body weight and feed intake of rats were recorded every 3 days. After feeding for 35 days, the serum was stored at −80°C for blood lipids detection. Colon fecal samples were collected and stored at −80°C for the detection of intestinal flora.

**TABLE 1:** Treatment for experimental rats.

Group	Diet	Treatment
NC (Normal control)	Normal diet	Sterile water (1 mL/100 g bw/day)
HM (High‐fat diet model)	High‐fat diet	Sterile water (1 mL/100 g bw/day)
PCI (Positive control I)	High‐fat diet	Simvastatin (6.7 mg/kg bw/day)
PCII (Positive control II)	High‐fat diet	Fenofibrate (16.7 mg/kg bw/day)
AAP (*Auricularia auricular* Polysaccharide)	High‐fat diet	AAP (100 mg/kg bw/day)
TFP (*Tremella fuciformis* Polysaccharide)	High‐fat diet	TFP (100 mg/kg bw/day)
HF (*Crataegus* Flavonoid)	High‐fat diet	HF (100 mg/kg bw/day)
LDC (Low‐dose combination)	High‐fat diet	AAP (25 mg/kg bw/day) + TFP (25 mg/kg bw/day) + HF (25 mg/kg bw/day)
MDC (Medium dose combination)	High‐fat diet	AAP (50 mg/kg bw/day) + TFP (50 mg/kg bw/day) + HF (50 mg/kg bw/day)
HDC (High‐dose combination)	High‐fat diet	AAP (100 mg/kg bw/day) + TFP (100 mg/kg bw/day) + HF (100 mg/kg bw/day)

### 2.3 Preparation and process optimization of polysaccharides and flavonoids

#### 2.3.1 Preparation of HF

Seventy percent ethanol was added into *Crataegus* powder in a certain proportion. The supernatant was extracted in a constant temperature water bath at 80°C for 20 min and then centrifuged at 860 ×*g* for 10 min to collect the supernatant. The supernatant was concentrated to a certain volume by rotary evaporation and refrigerated for later use.

#### 2.3.2 AAP extraction and optimization

Under each factor (ultrasonic time: 4, 6, 8, 10, and 12 min; ultrasonic temperature: 50, 60, 70, 80, and 90°C; material–liquid ratio: 1:30, 1:40, 1:50, 1:60, and 1:70), with the assistance of ultrasound (150 w), we selected five levels to optimize the single factor for polysaccharide extraction conditions of *A. auricula*. And then, (L_9_3^3^) orthogonal design was tested (Table 2).

**TABLE 2:** The orthogonal experiment factor level table.

Level	Factors		
	A ultrasonic temperature/°C	B ultrasonic time/min	C material–liquid ratio/(g/mL)
1	50	8	1:40
2	60	10	1:50
3	70	12	1:60

#### 2.3.3 TFP extraction and optimization

Under each factor (ultrasonic time: 20, 30, 40, 50, and 60 min; ultrasonic temperature:40, 50, 60, 70, and 80°C; material–liquid ratio: 1:30, 1:40, 1:50, 1:60, and 1:70), with the assistance of ultrasound (150 w), we selected five levels for a single‐factor experiment. According to the results of the single‐factor experiment, a response surface methodology experiment was carried out to obtain the optimal synchronous extraction parameters, taking the extraction rate of polysaccharide as the response value, and the ultrasonic temperature (A), ultrasonic time (B), material–liquid ratio (C) were independent variable (Table 3).

**TABLE 3:** The actual levels of the operational parameters and observed values of Box–Behnken design.

No.	A temperature/°C	B time/min	C material–liquid ratio/(g/mL)	Extraction yield/%
1	70	50	80	18.23
2	60	50	70	18.63
3	70	60	70	17.47
4	70	50	60	17.30
5	50	50	60	16.31
6	50	40	70	14.28
7	60	50	70	18.61
8	60	60	60	16.67
9	60	40	80	14.48
10	60	50	70	18.63
11	50	50	80	16.59
12	60	40	60	15.15
13	50	60	70	17.10
14	60	50	70	18.55
15	60	60	80	17.97
16	60	50	70	18.88
17	70	40	70	15.25

#### 2.3.4 Polysaccharide and flavonoid content

Polysaccharide contents were estimated by the phenol–sulfuric acid method using glucose as a standard (DuBois et al., 2002). Flavonoid content was measured by the aluminum nitrate colorimetric method (Wang et al., 2022). The polysaccharide extract is concentrated in a rotary evaporator. Then, four volumes of ethanol were added to precipitate the polysaccharides in the extract. The resulting precipitate was collected by centrifugation, washed with deionized water, and spun to remove the ethanol, and freeze‐dried for use. The component analysis is shown in Table S1. Flavonoid extract was concentrated in rotary evaporator for use. The component analysis is shown in Table S2.

### 2.4 Determination of monosaccharide composition by high‐performance liquid chromatography (HPLC)

Polysaccharide samples were treated with 4 mol/L TFA (trifluoroacetic acid), 0.5 mol/L PMP‐methanol solution, 0.3 mol/L NaOH solution, and 0.3 mol/L HCl solution, respectively, for HPLC detection. HPLC conditions: SHISEIDO C18 column (4.6 mm × 250 mm, 5 μm), mobile phase: A: 0.1 mol/L KH_2_PO_4_ (pH = 6.8); B: acetonitrile; A: B = 82: 18; flow rate: 1.0 mL/min; column temperature is 25°C; injection volume is 10 μL; wavelength is 245 nm.

### 2.5 Measurement of body weights, organ indexes, and fat index

During feeding, the body weights of rats were measured once weekly. The liver, spleen, heart, and kidney were weighed at the eighth week after the start of the feeding period. The following formula of organ index was determined (Guoying et al., 2017);

$$\text{Organ index}\;\left(\%\right)=\left[\text{organ weight}\;\left(g\right)/\text{body weight}\;\left(g\right)\right]\times 100\%$$

Fat index: the fatty tissues around the kidney and genitalia were dissected and weighed and the fat index was calculated. The formula was as follows.

$$\mathrm{fat}\;\text{index}\;\left(\%\right)=\left[\mathrm{fat}\;\mathrm{wet}\;\text{weight}\;\left(g\right)/\text{Weight}\;\left(g\right)\right]\times 100\%$$

### 2.6 Biochemical analysis

TC, TG, HDL‐C, LDL‐C, ALT, and AST were tested by ELISA kits (Nanjing Jiancheng Bioengineering Institute), and the arteriosclerosis index (AI) was calculated.

$$\mathrm{AI}=\left[\mathrm{TC}-\left(\mathrm{HDL}-C\right)\right]/\left(\mathrm{HDL}-C\right)$$

### 2.7 Observation of the pathological changes of hepatic tissue

The liver of the same part (hepatic lobule) of the rat soaked in 4% paraformaldehyde fixative was taken, and then dehydrate → paraffin embedding → section → HE staining → optical microscope to observe whether the liver tissue structure is degenerated (Blank et al., 2019).

### 2.8 Analysis of intestinal flora by high‐throughput sequencing

Colon fecal samples were collected from rats under sterile conditions. Microbial DNA was extracted from NC, HM, and HDC groups using the EZNA®stool DNA Kit (Omega Bio‐Tek) according to the manufacturer's protocols. The V3–V4 region of the bacteria 16S rRNA gene was amplified by PCR (94°C for 5 min, followed by 28 cycles at 94°C for 30 s, 55°C for 30s and 72°C for 60 s, a final extension at 72°C for 7 min) using primers 338F (5′‐ACTCCTACGGGAGGCAGCAG‐3′) and 806R (5′‐GGACTACNNGGG TATCTAAT‐3′). PCR reactions were performed in triplicate (Wang et al., 2016), 25‐μL mixture containing 1 μL of forward primer (5 μM), 1 μL of reverse primer (5 μM), 3 μL of BSA (2 ng/μL), 12.5 μL of 2xTaq Plus Master Mix, and 7.5 μL of ddH_2_O.

Sequencing amplicons were extracted from 1% agarose gels and purified using the AxyPrep DNA Gel Extraction Kit (Axygen Biosciences) (Vargas‐Albores et al., 2017) according to the manufacturer's instructions. Purified amplicons were pooled in equimolar and paired‐end (PE) sequenced (2 × 300) on an Illumina MiSeq platform according to the standard protocols.

Paired‐end sequencing was performed using the Illumina MiSeq platform to remove primer mismatches, sequences <150 bp in length, and ambiguous bases. The OTU similarity was set to 97%. According to the Silva database, the species classification information of each OTU was determined, and alpha diversity analysis via Mothur software (including Shannon, ACE, and Chao1; Cui et al., 2017).

### 2.9 Statistical analysis

The data were analyzed using SPSS software (version 11.5, SPSS) and *p* < .05 was considered as statistically significant. All the data were presented as mean ± SD.

## 3 RESULTS

### 3.1 Optimization of the polysaccharide extraction process

#### 3.1.1 Polysaccharide extraction from *Auricularia auricular*

After a series of single‐factor experiments, we obtained the extraction conditions of polysaccharides from *A. auricula* and the results of the orthogonal experiments (Table 4).

**TABLE 4:** Orthogonal test results of ultrasonic‐assisted extraction of AAP.

Run	Factor			Extraction yield/%
	A/°C	B/min	C/(g/mL)	
1	1	1	1	10.84
2	1	2	2	10.13
3	1	3	3	9.90
4	2	1	2	13.25
5	2	2	3	11.36
6	2	3	1	10.73
7	3	1	3	9.88
8	3	2	1	10.38
9	3	3	2	10.08
K_1_	30.87	33.97	31.95	–
K_2_	35.34	31.87	33.46	–
K_3_	30.34	30.71	31.14	–
k_1_	10.29	11.32	10.65	–
k_2_	11.78	10.62	11.15	–
k_3_	10.11	10.24	10.38	–
R	1.67	1.09	0.77	–
Primary and secondary order	A﹥B﹥C			–
Optimal level	A_2_	B_1_	C_2_	–
Optimal combination	A_2_B_1_C_2_			–

The results of range analysis were A > B > C, which indicated that among the factors that affect the extraction yield of crude polysaccharides from *A. auricula*, the ultrasonic temperature has the most influence on the results, followed by the ultrasonic time, and the material–liquid ratio has the least influence. The optimal extraction process combination obtained by optimization is A_2_B_1_C_2_: the ultrasonic temperature is 60°C, the ultrasonic time is 8 min, and the ultrasonic material–liquid ratio is 1:50. The average yield of crude polysaccharides from *A. auricula* was 13.3%, which was higher than the results of Cai et al. (2015).

#### 3.1.2 Polysaccharide extraction from tremella

After a series of single‐factor experiments, we obtained the extraction conditions of polysaccharides from *Tremella* and the corresponding results are shown in Table 3.

The analysis of variance for the experimental results of the Box–Behnken design is presented in Table 5. The total return value *F* = 91.90, *p* < .0001, indicating that the total regression was statistically difference. The correction index R^2^ (Adj) = 98.08%, and the index of variation C.V.% = 1.24, the equation of the fitting model is highly reliable. In summary, the model equation can be used to predict and analyze the extraction conditions of *Tremella* polysaccharide. The results also showed that the influence of each factor is B > A > C, that is, time > temperature > material–liquid ratio.

**TABLE 5:** ANOVA for the effect of temperature, time, and material–liquid ratio on the extraction yield using the response surface model.

Source	Sum of squares	df	Mean square	*F*‐value	*p*‐Value	Significance
Model	37.22	9	4.14	91.90	<.0001	**
A	1.97	1	1.97	43.78	.0003	**
B	12.63	1	12.63	280.58	<.0001	**
C	0.42	1	0.42	9.41	.0181	*
AB	0.09	1	0.09	2.00	.2002	–
AC	0.10	1	0.10	2.35	.1694	–
BC	0.97	1	0.97	21.56	.0024	**
A^2^	2.68	1	2.68	59.51	.0001	**
B^2^	14.22	1	14.22	315.95	<.0001	**
C^2^	2.40	1	2.40	53.34	.0002	**
Residual	0.31	7	0.04	‐	–	–
Lack of Fit	0.25	3	0.08	5.15	.0737	Not significant
Pure Error	0.06	4	0.02	–	–	–
Cor Total	37.53	16	–	–	–	–

*Note*: * is extremely significant (*p* < .05), ** is extremely significant (*p <* .01).

**FIGURE 1:**
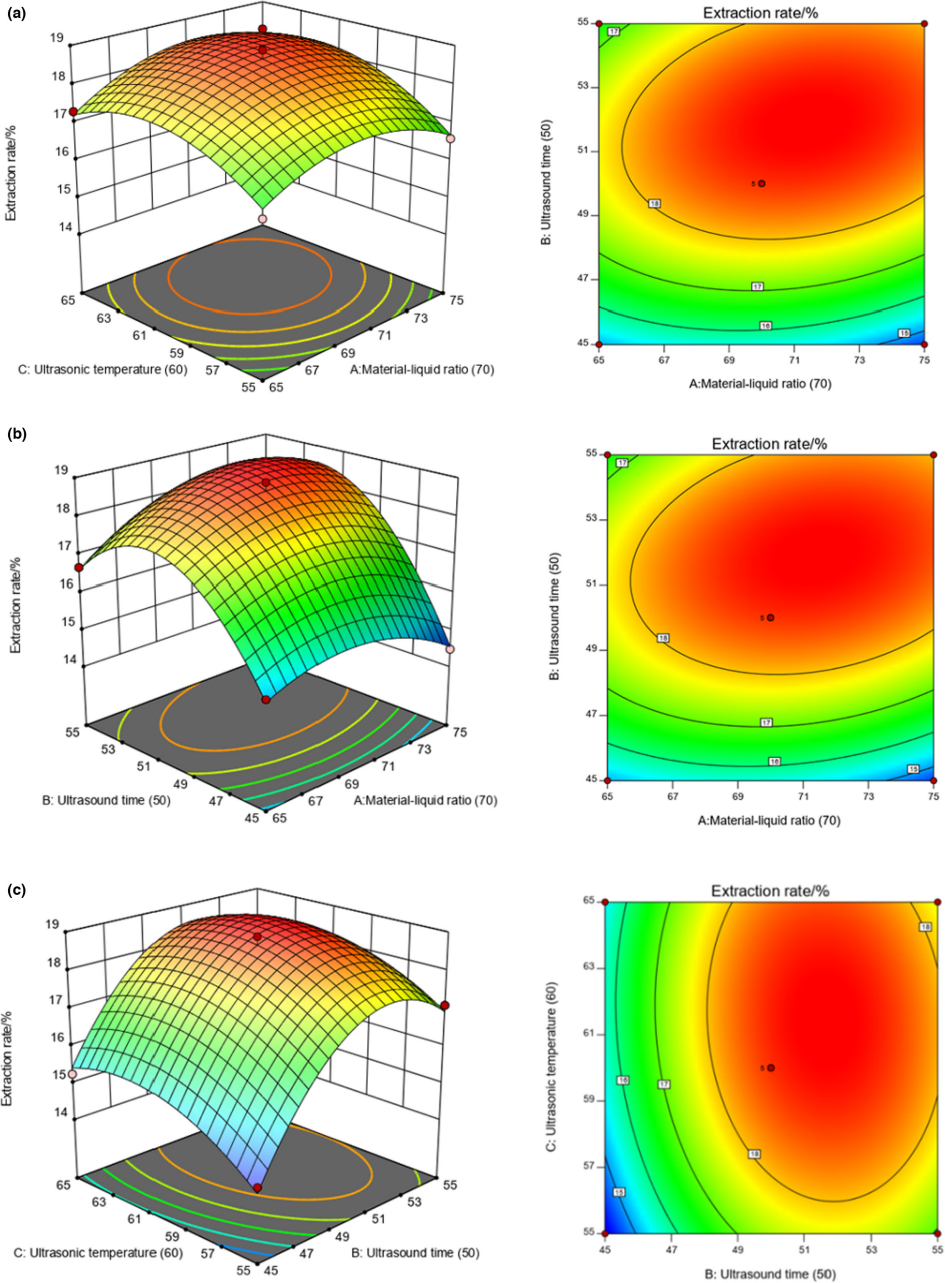
Response surface map and contour map of the interaction of two factors on the extraction of TP. (a) Material–liquid ratio and ultrasonic temperature; (b) Material–liquid ratio and ultrasonic time; (c) Ultrasound time and ultrasonic temperature.

By comparing the response surface analysis chart and the contour map (Figure 1), it can be seen that the interaction between the two factors of ultrasonic time (B) and material–liquid ratio (C) has a significant effect on the extraction of TFP, while in other factors, the interaction between the two has less effect on the extraction of TFP.

According to the typical analysis data, the optimal extraction process conditions, ultrasonic time 53.70 min, ultrasonic temperature 63.00°C, and material–liquid ratio 1:73 were carried out. The maximum expected extraction rate of TFP reached 19.00%, which is in line with theoretical predictions, and the values are similar. This is not much different from the results of Zou and Hou (2017).

### 3.2 Monosaccharide composition analysis

**FIGURE 2:**
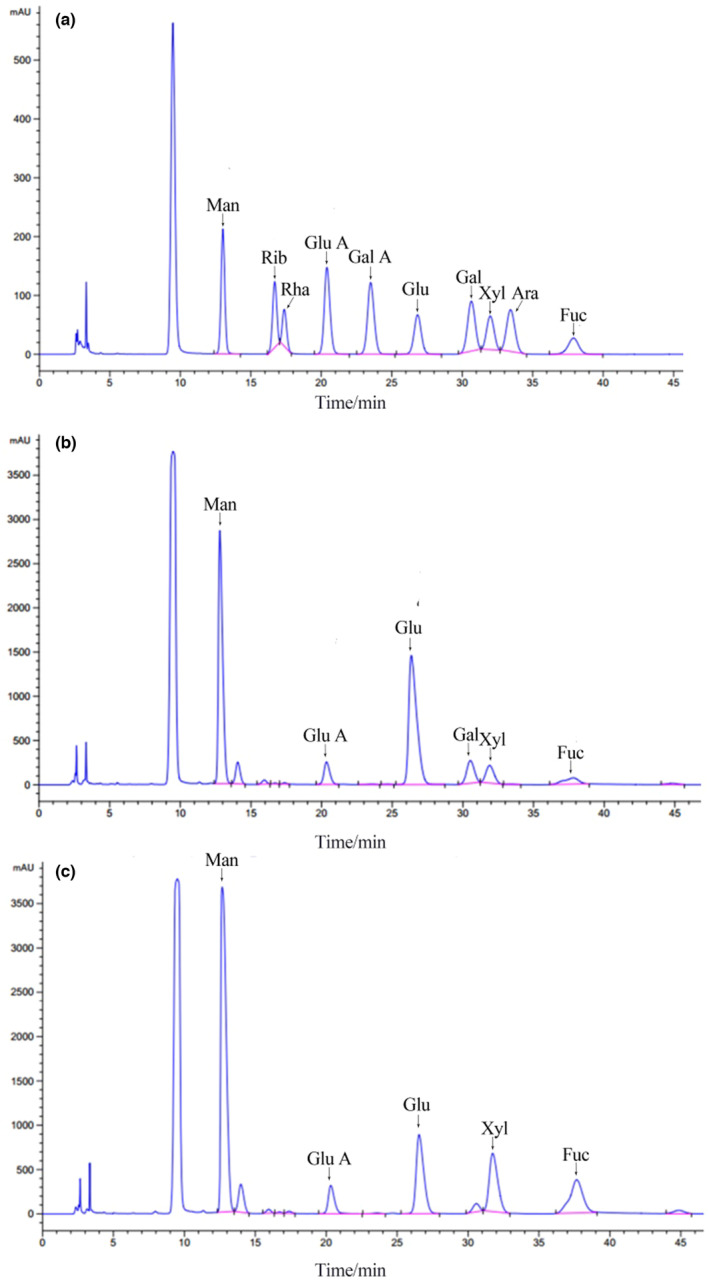
Monosaccharides of polysaccharides from *A. auricula* and *Tremella* (Man: Mannose; Rib: Ribose; Rha: Rhamnose; Glu A: Glucuronic acid; Gal A: Galacturonic acid; Glu: Glucose; Gal: Galactose; Xyl: Xylose; Ara: Arabinose; Fru: Fructose). (a) monosaccharide standard; (b) monosaccharides of AAP; (c) monosaccharides of TFP.

The monosaccharides of polysaccharide from *A. auricula* and *Tremella* were analyzed. The results showed the monosaccharide composition of AAP, mannose: glucuronic acid: glucose: galactose: xylose: fucose (8.64: 1.00: 13.47: 1.69: 2.13); The monosaccharide composition of TFP was as follows: mannose: glucuronic acid: glucose: xylose: fucose (10.86: 1.00: 5.89: 5.59: 6.68; Figure 2).

### 3.3 Effect of polysaccharide and flavonoid on the body weights, organ indexes, and fat index

**FIGURE 3:**
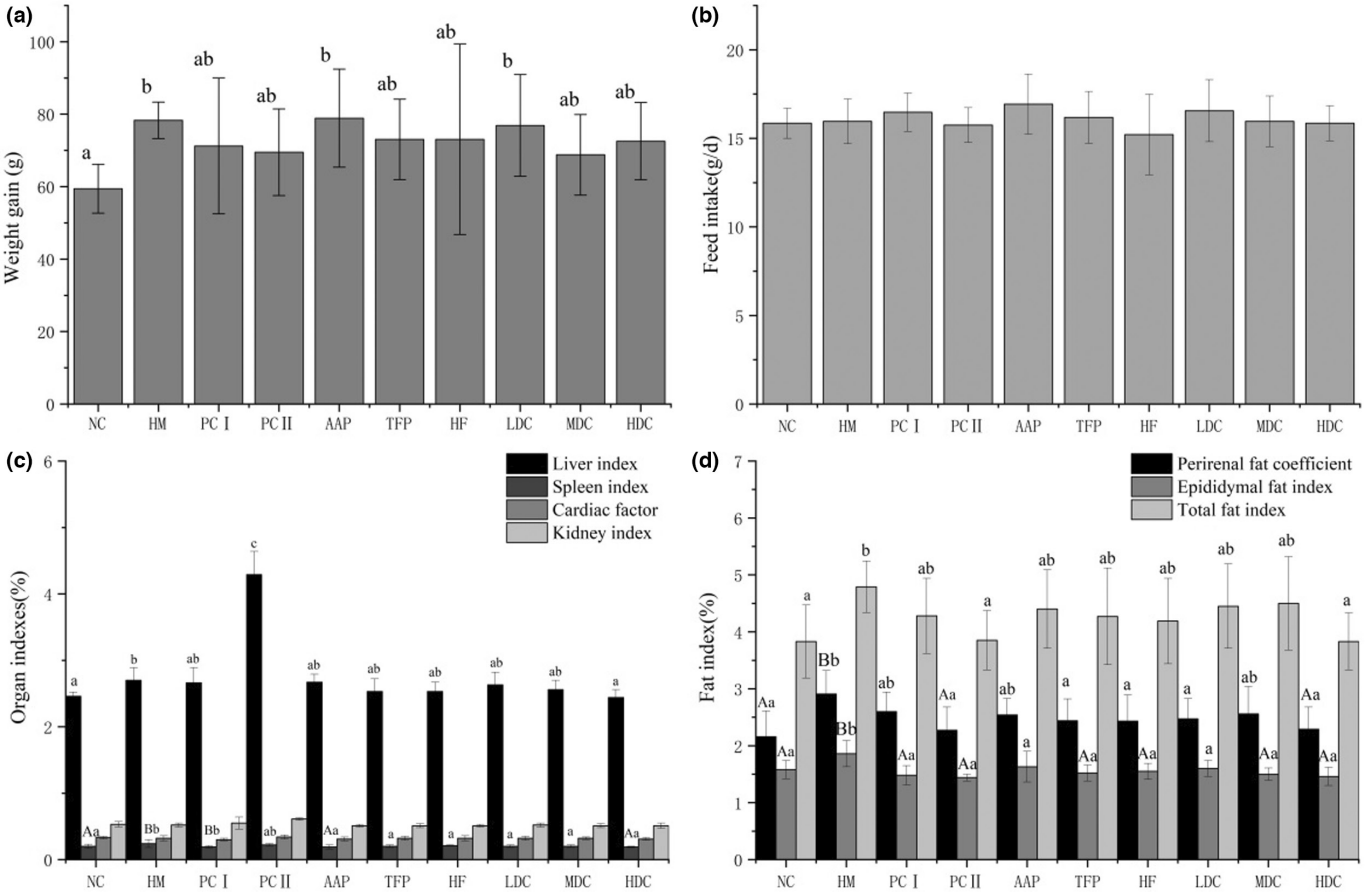
The main index of rats. (a) weight gain; (b) food intake; (c) organ index; (d) fat index. The data are presented as the means ± SD (*n* = 9). Different lowercase letters in the same chart represent significant differences between different treatments (*p* < .05), and different capital letters represent significant differences (*p* < .01).

As shown in Figure 3a, compared with the NC group, the increase in body weight in the HM group was significantly higher (*p* < .05), indicating that long‐term consumption of a high‐fat diet will cause a significant increase in the body weight of rats. This result is consistent with the studies of Zou et al. (2016). But the body weight of experimental rats did not increase significantly compared with NC or HM except AAP and LDC which were the same as HM. There was little difference in feed intake between the groups (Figure 3b).

High‐fat diets cause liver and spleen damage in rats, and liver and spleen index increased. Compared with the NC group, liver index in the HM group was increased, and the difference was significant (*p* < .05). Compared with the HM group, HDC group's liver index decreased by 9.6% (*p* < .05) and spleen index decreased by 20.8% (*p* < .01), which are shown in Figure 3c. As the dose of polysaccharides and flavonoids increased, the liver indexes gradually decreased, showing a dose dependent. There are no significant differences in the heart and kidney indexes of the rats in each group in this test. The visceral damage of the rats in each group is not serious, which is consistent with the results of Chen et al. (2018).

As shown in Figure 3d, compared with the HM group, the HDC group had a reduction effect on the perirenal fat coefficient, which decreased by 21.3% (*p* < .01), TFP, HE, and LDC groups also had a reduction effect on perirenal fat index (*p* < .05). Compared with the HM group, only the HDC group had the most obvious effect of reducing the total fat coefficient, which decreased by 21.5% and had statistical significance (*p* < .05). Thus, it can be explained that the HDC group may have an effect of fat index loss (Burke et al., 2018).

### 3.4 Effect of polysaccharide and flavonoid on serum biochemical parameters

**FIGURE 4:**
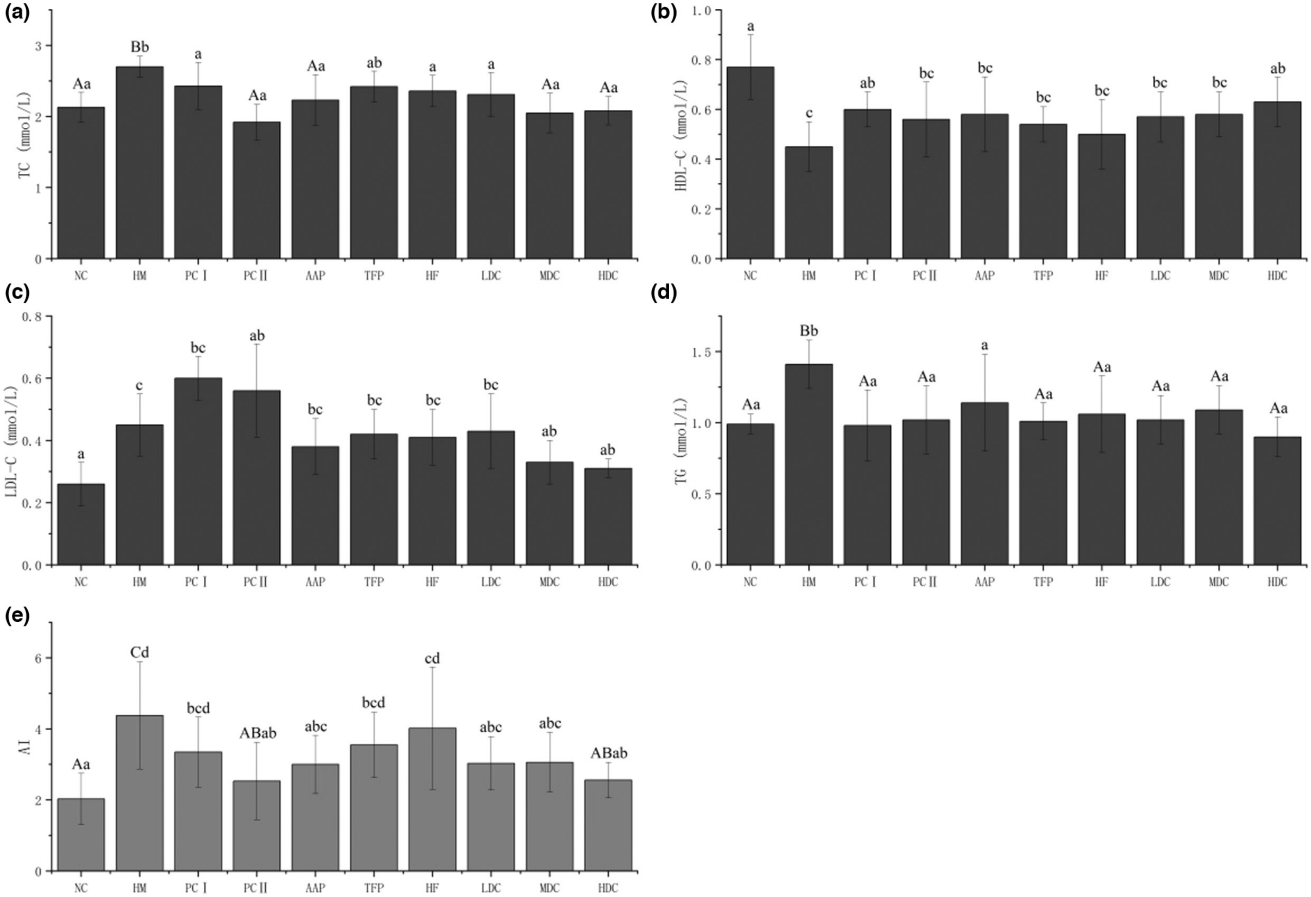
Effects of polysaccharides and flavonoid extracts on blood lipid levels in rats. (a) TC levels in serum; (b) HDL‐C levels in serum; (c) LDL‐C levels in serum; (d) TG levels in serum; (e) the arteriosclerosis index (AI).

The TC and TG levels of AAP, TFP, HF, LDC, MDC, and HDC groups were decreased significantly compared with HM group as shown in Figure 4a–e. While this phenomenon was not maintained for LDL‐C levels except MDC (0.33 ± 0.07 mmol/L) and HDC (0.31 ± 0.03 mmol/L) groups, high level of HDL‐C in the blood has been known as an effective factor in blood lipids regulation (Han et al., 2018). In the research, high dose of polysaccharide and flavonoid played an important role on HDL‐C significant increase (*p* < .05) of the rats in HDC group (0.63 ± 0.10 mmol/L) which was compared with HM group (0.45 ± 0.10 mmol/L).

With the regulation of blood lipids in rats, the average value of AI of rats in each group showed different changes. The larger the value of AI, the higher the risk of atherosclerotic (Karjalainen et al., 2020). The value of the HM group is as high as 4.38 ± 1.51, which has a higher risk of atherosclerosis. After drug intervention, the AI values of rats in the LDC, MDC, and HDC groups decreased by 30.8%, 30.1%, and 41.6, respectively. In the polysaccharide and flavonoid group, only the AAP group showed a significant decrease, but there was no significant difference between the HF group and the THP group.

### 3.5 Effects of polysaccharide and flavonoid on hepatic biochemical indicator

**FIGURE 5:**
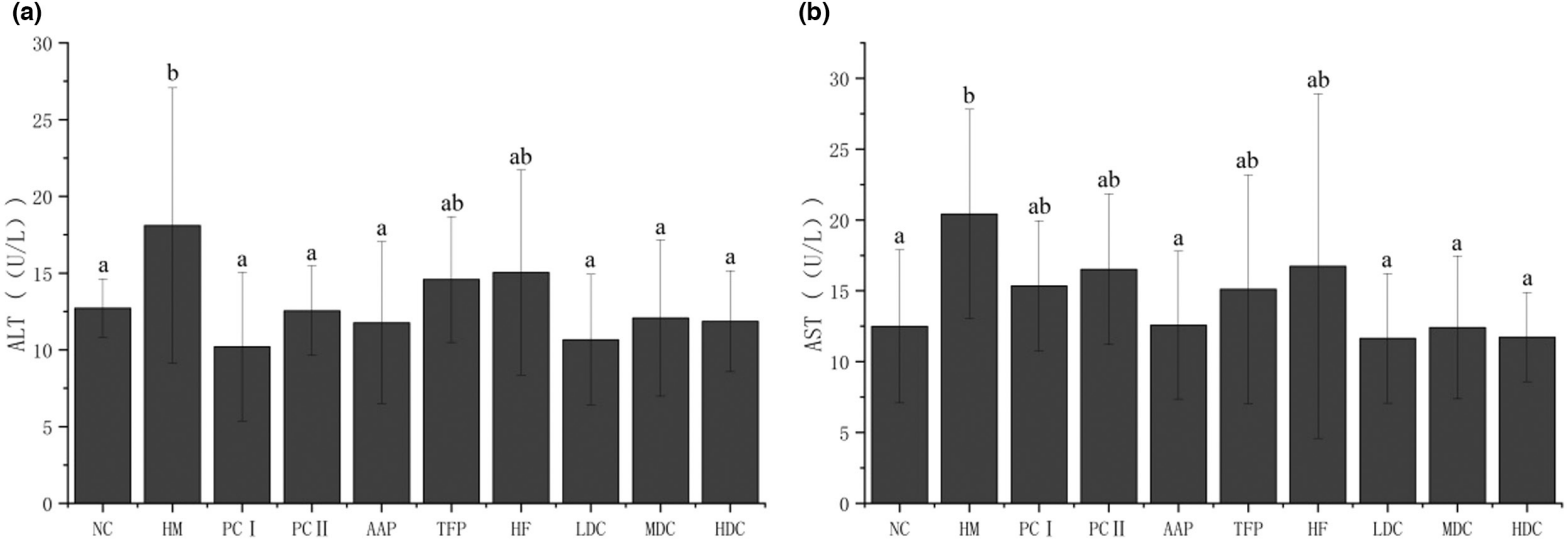
The effect of polysaccharide and flavonoid extract on the activity of ALT and AST in rat serum. (a) ALT index; (b) AST index.

As shown in Figure 5, the AST and ALT enzyme activities in the serum of AAP LDC, MDC, and HDC group were significantly reduced (*p* < .05) compared with HM group. This effect was even better than PCI and PCII groups on AST level. However, TFP group and HF group were not reduced significantly different (*p* > .05), indicating that the combined use of the AAP and HF extract has a protective effect on the liver health of hyperlipidemia rats. This result of flavonoids and polysaccharides is consistent with the treatment of hepatitis (Du et al., 2017).

### 3.6 Histomorphological analysis of liver tissue

**FIGURE 6:**
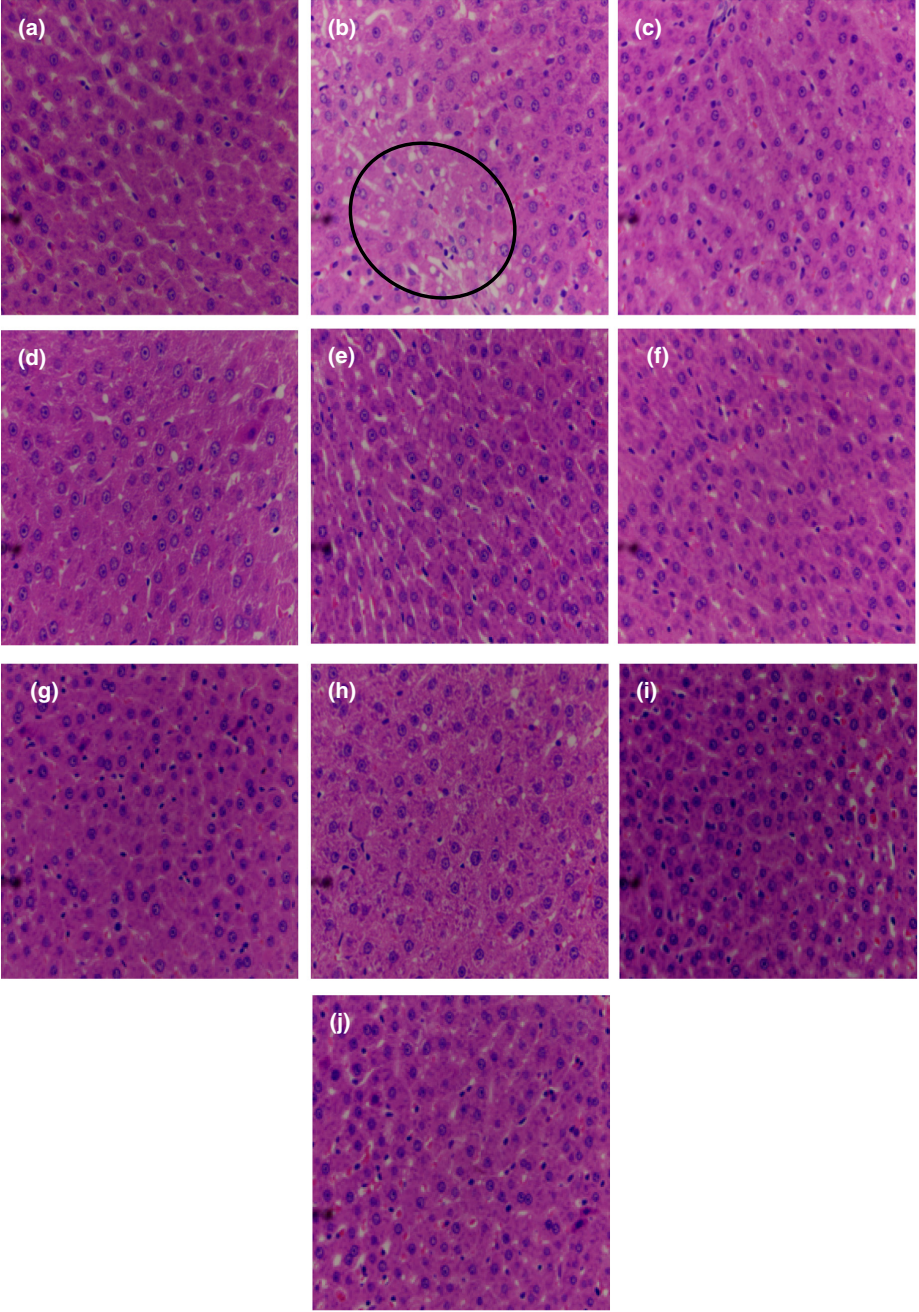
HE staining analysis of liver tissue (×400). (a) NC; (b) HM; (c) PC I; (d) PC II; (e) AAP; (f) THP; (g) HF; (h) LDC; (i) MDC; (j) HDC.

The healthy liver tissue structure was clear and the hepatocytes were arranged in a cord‐like manner (Beckwitt et al., 2018). At the same time, the hepatocyte nuclei were centered, the cytoplasm was abundant, and the cell membrane was clear as shown in Figure 6a. No fatty vacuole cell nuclei were found in the center of the cells. Hepatic sinus was significantly narrowed in the model group, hepatic cords were arranged disorderly, and fatty vacuolar nuclei of different sizes appeared in some hepatocytes. The liver tissue structure became clearer than that in the model group after administration. The intervention effect was most obvious in the MDC and LDC groups (Figure 6).

### 3.7 Effects of polysaccharide and flavonoid on intestinal flora

#### 3.7.1 Comparison of intestinal flora at OTU level in rats

**FIGURE 7:**
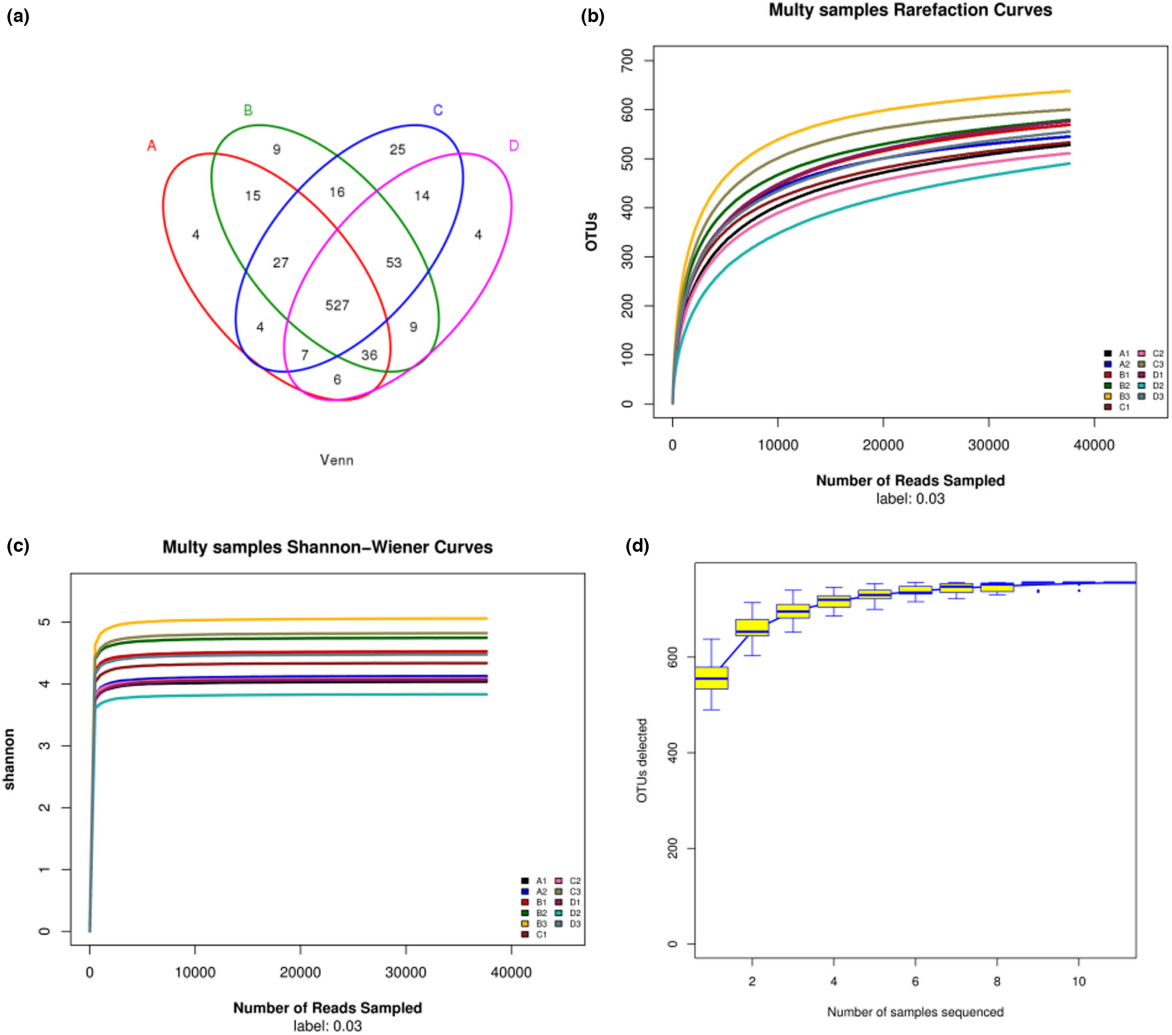
Alpha diversity curve. (A1, A2: NC group; B1, B2, and B3: HM group; C1, C2, and C3: PCI group; D1, D2, and D3: HDC group) (a) Venn diagram of OUT; (b) Rarefaction curves; (c) Shannon–Wiener curves; (d) Species accumulation curves.

The total number of taxonomic units (OTUs) was 6124. As shown in Figure 7a, the Venn diagram was used to evaluate the distribution of OTUs among the different samples. We obtained 626, 692, 673, and 656 OTUs from NC, HM, PCI, and HDC groups, respectively.

#### 3.7.2 Alpha diversity analysis

Alpha diversity can reflect the abundance and diversity of the microbial community (Wagner et al., 2018). The sample volume in our study was relatively large enough to reflect the species richness (Figure 7b–d). The intestinal microecological diversity of the HDC group was significantly lower than that of the HM group (*p* < .05) as shown in Table 6. The richness of the intestinal flora and the diversity of the microbial community in the HDC group were close to those in the NC group.

**TABLE 6:** Alpha Diversity Index.

Group	Chao1	Observed species	PD whole tree	Shannon
NC	594.65 ± 11.50	536.50 ± 12.02	41.46 ± 0.67	5.89 ± 0.09a
HM	645.60 ± 31.94	595.33 ± 37.29	43.20 ± 2.29	6.89 ± 0.39b
PCI	600.13 ± 29.72	547.93 ± 46.32	41.14 ± 3.06	6.37 ± 0.54ab
HDC	626.77 ± 27.09	540.33 ± 44.84	40.57 ± 2.43	5.95 ± 0.47a

#### 3.7.3 Beta diversity analysis

**FIGURE 8:**
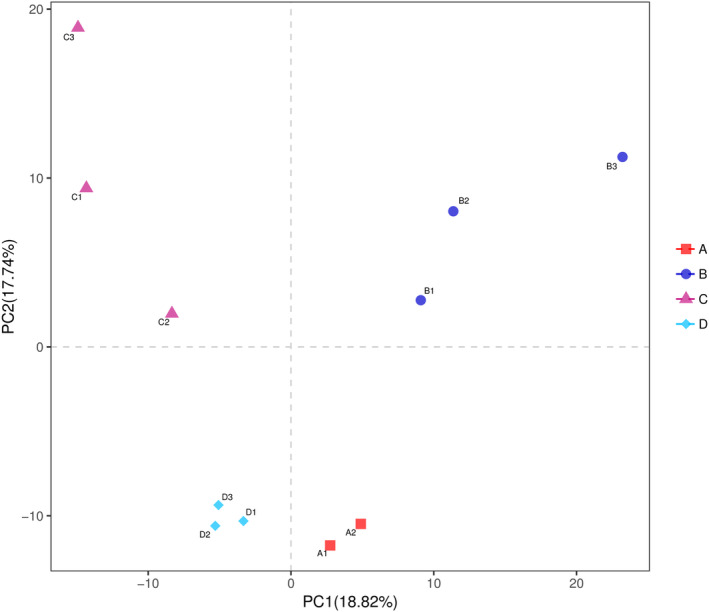
Bate diversity curve of intestinal microorganisms in rats. (A1, A2: NC group; B1, B2, and B3: HM group; C1, C2, and C3: PCI group; D1, D2, and D3: HDC group).

Beta diversity analysis is mainly to investigate the difference in community structure among different samples. As shown in Figure 8, the contribution rates of the first principal component and the second principal component based on PLS‐DA analysis were 18.82% and 17.74%, respectively. Due to different treatment methods, the intestinal flora of the four groups of rats was significantly different and clustered into four categories. The results showed that the distance between the NC and the HDC groups was small, indicating that the degree of difference between the two groups of samples was small, while the distance between the PCI group and the HM group was large, indicating the difference between the two groups of samples. In conclusion, the HDC group effectively improved the composition of intestinal flora, making it more similar to the NC group.

#### 3.7.4 Microbial community structure at the phylum and genus level

**FIGURE 9:**
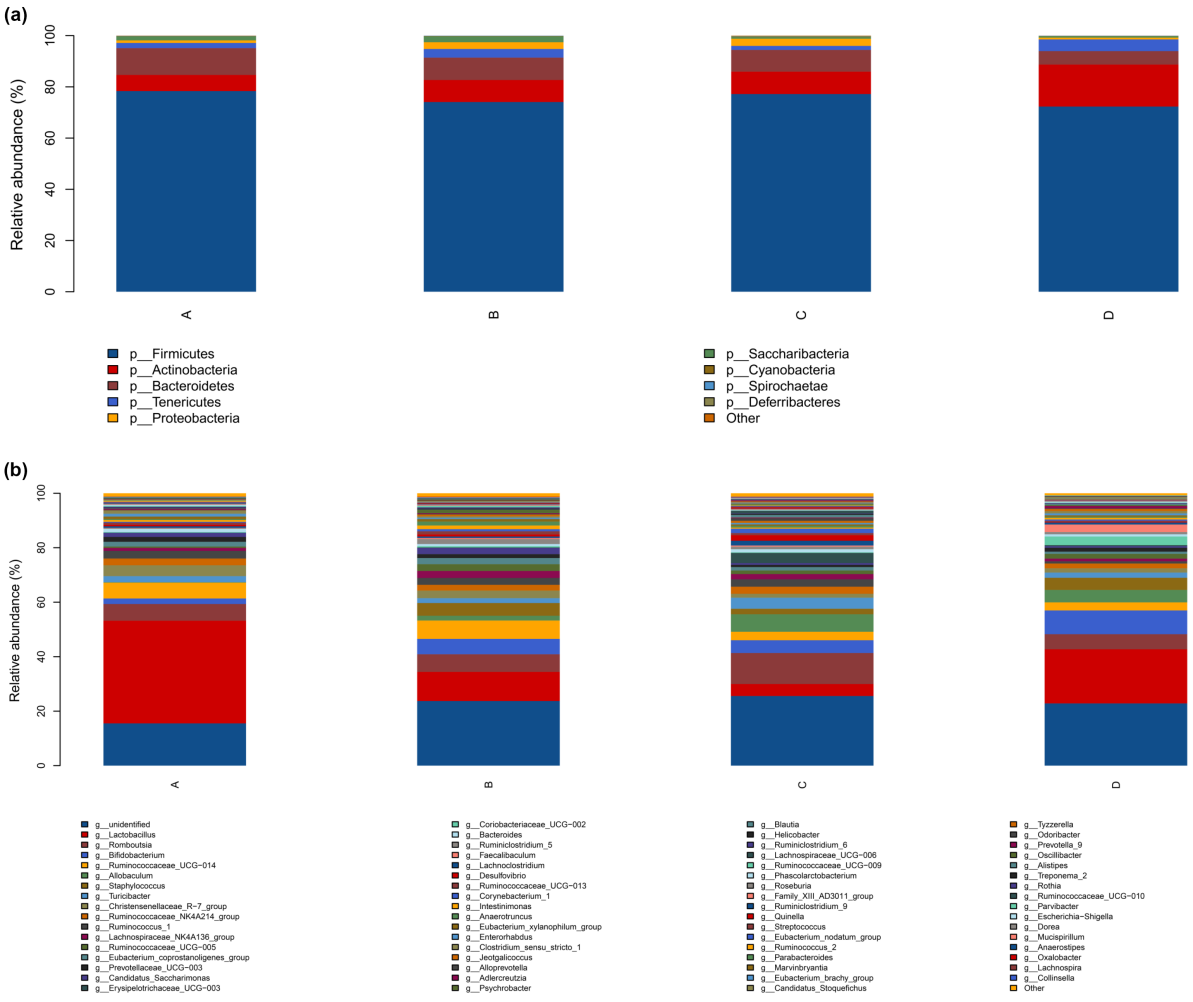
Species composition analysis bar chart (A: NC group; B: HM group; C: PCI group; D: HDC group).

The classification of sequences from the samples resulted in nine different phyla that were identified in this study (Figure 9a). After treated with HDC, the *Actinomycetes* in the rat's intestine of HDC group were significantly higher than the HM group (16.41% vs. 8.6%, *p* < .05), while the abundance of *Bacteroides* decreased compared with the NC group and model group, but there was no significant difference. At the genus level, the top 49 genera are listed in Figure 9b. Compared with the HM group, five kinds of microorganisms of the HDC group were reduced significantly such as *Alistipes* (0.14% vs. 0.04%, *p* < .05), *Helicobacter* (0.40% vs. 0.06%), *Desulfovibrio* (0.74% vs. 0.24%), *Dorea* (0.02% vs. 0.13%), and *Escherichia coli/Shigella* (0.12% vs. 0.01%).

#### 3.7.5 LefSe analysis of rat intestinal microbial species

**FIGURE 10:**
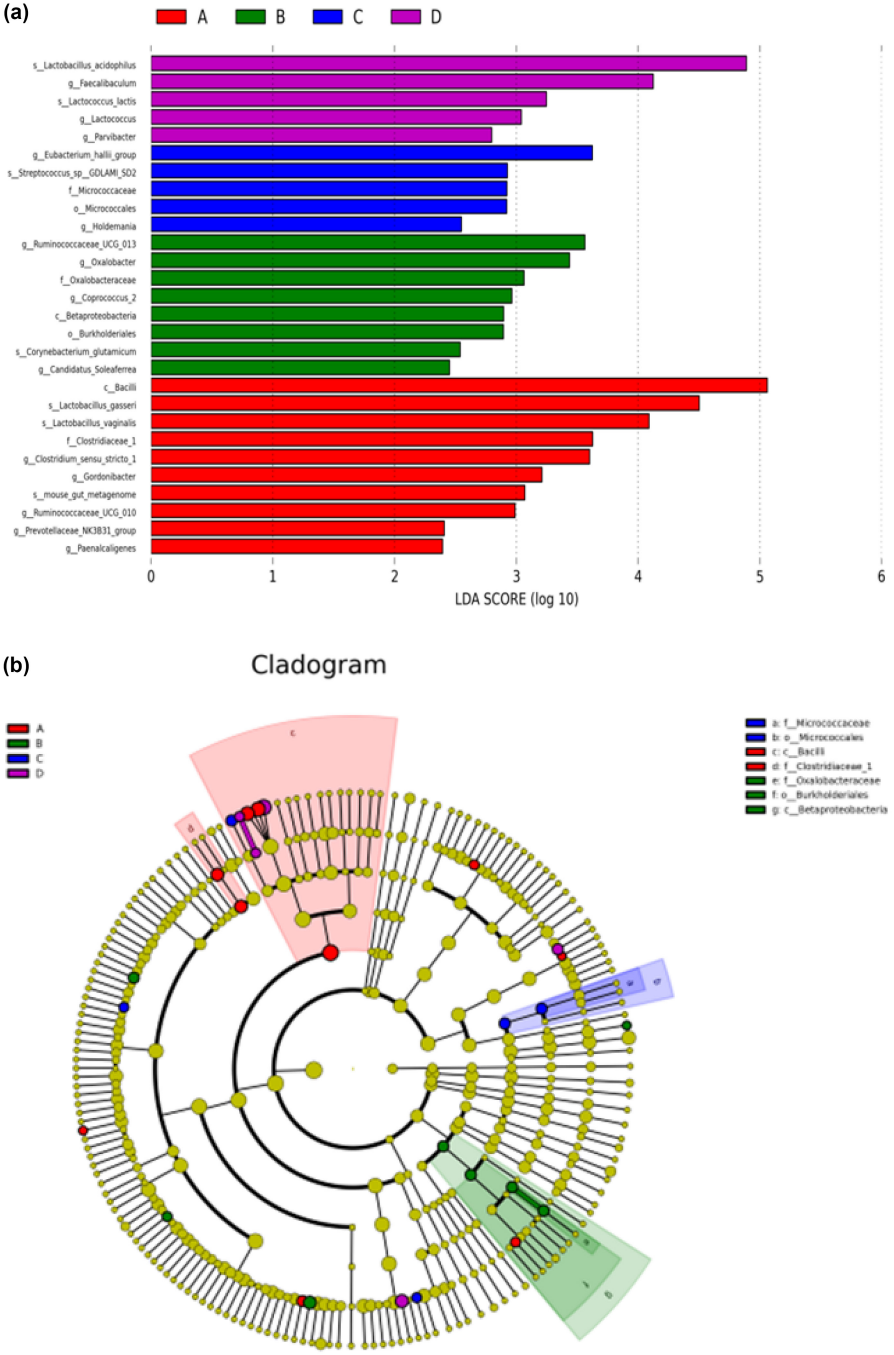
(a) Histogram of intestinal microorganism LDA distribution in four groups of rats; (b) Intestinal microbial evolution branch of four groups of rats. (A: NC group; B: HM group; C: PCI group; D: HDC group).

According to linear discriminative analysis (LDA), LefSe was performed to obtain the cladogram representation and the predominant bacteria in the intestinal microbiota in the four groups as shown in Figure 10a, the abundance of species in different groups was different. The evolutionary branch diagram of the four groups of samples (Figure 10b), from outside to inside, represents the subordinate to door level classification (Cox et al., 2014). *Bacillus*, *Clostridiaceae* 1, and *Clostridium* sensu stricto 1 were enriched in the NC group, *β‐proteobacteria*, *Burkholderia*, *Oxalobacteraceae*, and *Ruminococcaceae* UCG 013 were enriched in the HM group, *Micrococcus*, *Micrococcaceae*, *Eubacterium cholerae*, *Holdemanella*, and *Streptococcus* sp *GDLAMI SD* were enriched in the PCI group, whereas *Fecal bacillus*, *Lactococcus*, *Bacillus pectineus*, *Lactobacillus acidophilus*, and *Lactococcus lactis* were enriched in the HDC group.

## 4 CONCLUSION AND DISCUSSION

Natural polysaccharides distribute in different parts of cells, and the degree of ease of extraction varies (Wang et al., 2021). In this study, nitrogen freezing pulverization was chosen before *A. auricular* polysaccharides and *Tremella* polysaccharide extraction with ultrasound assisted. The composition of *A. auricula* polysaccharide is different from Zeng et al. (2012) and the composition of *Tremella* polysaccharide is different from Zou and Hou (2017). Up to now, there are still great discrepancies about structural properties of *A. auricular* polysaccharides and *Tremella* polysaccharides, including the molecular weights, monosaccharide compositions, and glycosidic linkages (Shi et al., 2023; Zhang et al., 2022). The study found that the different chemical modification methods for monosaccharides resulted in slightly different ingredients.

In recent years, the incidence of hyperlipidemia has been increasing year by year. Statins are widely used in clinical practice, but they have certain adverse reactions. Some people may have muscular toxicity after taking statins, and rhabdomyolysis occurs in severe cases (He et al., 2018). Edible fungi polysaccharides can improve lipid metabolism disorders mainly by inhibiting or promoting the expression of related lipid metabolism enzymes and related factors, improving the activity of bile acid synthesis, regulating intestinal flora, and alleviating oxidative stress reaction (Yin et al., 2020; Zhao et al., 2023). Flavonoids can promote the decomposition of cholesterol and triglyceride to improve liver function and lipid metabolism (Huiming et al., 2022; Lanlan et al., 2022). In order to evaluate the synergistic effects of polysaccharide and flavonoid, the hyperlipidemia model was established. Feeding with high‐fat diet, the liver index and spleen index increased significantly, while the liver index of the PCII group increased more obviously (*p* < .01) compared with NC group, which indicated that the lipid‐reducing medicine had a significant influence on the liver organ and reduced blood lipids in the meanwhile. This result was consistent with Ahmad et al. (2017). So, it is important to protect liver during the blood lipids regulation. In this study, the HDC had a good effect on lipid decrease such as TC and TG, and reduced the incidence of hyperlipidemia significantly. At the same time, the livers of the HDC group were more healthy than that in the HM group or in the PCII group. But the protective effects of TFP alone and HF alone on the livers of hyperlipidemia rats were not significant.

Intestinal flora diversity is the basis of promoting nutrient absorption and maintaining immunity metabolism and plays an important role in the regulation of lipid metabolism. High‐fat diet will lead to a decrease of intestinal flora diversity, a change in membrane integrity, an increase of permeability and lipopolysaccharide displacement, a change in immune system, the generation of inflammation and damage to the intestinal barrier (Netto Candido et al., 2018). Therefore, protecting the structure of intestinal flora is conducive to improving hyperlipidemia. Wang et al. (2014) found PAM (polysaccharide *Atractylodes macrocephala*), active polysaccharides, could improve and regulate the intestinal flora which was used as an oral adjuvant for intestinal disorder flora. Kaakoush and Morris (2017) found that in the effects of flavonoids, intestinal flora is considered to play a central role, not only because some flavonoids have bacteriostatic effects, but also promote the proliferation of some probiotics. The interaction between polysaccharides and flavonoids may change the absorption, distribution, and metabolism of polysaccharides in gastrointestinal digestion, and even change intestinal flora (Luo et al., 2009). In this study, Feeding high‐fat diet damaged the intestinal flora structure, increased the harmful bacteria such as *Escherichia coli/Shigella*, *Helicobacter*, *β‐Proteobacteria*, *Burkholderia*, *Clostridium*, and *Coprococcus*, and decreased the relative abundance of beneficial bacteria such as *Bifidobacteria* and *Lactobacillus*. *Clostridium* is a large and important group of bacteria in *Firmicutes*, containing a variety of intestinal pathogenic bacteria, which can produce exotoxins and have strong toxic effects on both humans and animals (Kociolek & Gerding, 2016). In the metabolic process, the increase of *Clostridium* will accelerate fat deposition, which leads to obesity (Turnbaugh et al., 2006). Harmful bacteria such as *Escherichia coli/Shigella*, *Clostridium* spp., *Helicobacter*, and *Vibrio desulfuricans* decreased, while beneficial bacteria such as *Lactobacillus* and *Bifidobacterium* increased in the HDC group. *Lactobacillus* and *Bifidobacterium* can regulate host metabolism and balance it, while *Lactobacillus* can further improve the structure of intestinal flora, participate in cholesterol metabolism and lipid regulation, and other diseases. In addition, *Lactobacillus* can inhibit intestinal pathogens and improve the intestinal environment. According to our results, the compound of polysaccharides and flavonoids showed synergistic effects on the regulation of intestinal flora for hyperlipidemia rats.

In summary, the experiment results verify the correctness of the hypothesis. The present study considers the effects of polysaccharide and flavonoid on blood lipids level and intestinal flora. The application of liquid nitrogen freezing crushing combined with ultrasonic‐assisted extraction technology can achieve a good extraction effect of polysaccharide, and the combination of polysaccharide and flavonoid can achieve a good reduction of blood lipids and protection of the liver, as well as a good positive regulation of intestinal flora. Flavonoids can effectively regulate the metabolism of lipids, and remove oxygen‐free radicals (Duan et al., 2021). Polysaccharides may regulate blood lipid levels by improving liver function, increasing short‐chain fatty acid content, and regulating intestinal flora structure in rats with high‐fat diet, and play a certain preventive role in the occurrence of hyperlipidemia. (Ge et al., 2022; Kadnikova et al., 2015; Qihuan et al., 2022). The synergy of polysaccharides and flavonoids is worth studying in the future. This study will play an important role in functional food production of polysaccharide and flavonoid in the future.

## AUTHOR CONTRIBUTIONS

**Ke Shi:** Conceptualization (equal); data curation (lead); methodology (equal); software (equal); validation (equal); writing – original draft (lead). **Tao Zhou:** Conceptualization (equal); data curation (equal); methodology (equal); software (equal); validation (equal). **Yu‐fei Yuan:** Conceptualization (equal); data curation (equal); methodology (equal); software (equal). **Dan‐dan Li:** Investigation (equal); resources (equal); supervision (equal). **Bin‐bin Gong:** Investigation (equal); resources (equal); supervision (equal). **Shan Gao:** Project administration (equal); resources (equal); supervision (equal). **Qi–jia Chen:** resources (equal); writing – review and editing (equal). **Yan‐dong Li:** Investigation (equal); project administration (equal); resources (lead). **Xue Han:** Formal analysis (equal); funding acquisition (equal); project administration (equal); resources (equal); supervision (equal); writing – review and editing (equal).

## FUNDING INFORMATION

The present work was supported by grants from the Key Research and Development Program of Hebei Province (No. 21327125D), the Science and Technology Plan Program of Shijiazhuang (No. 221500052A) and the National Natural Science Foundation of China (No. 22001056).

## CONFLICT OF INTEREST STATEMENT

The authors declare that there are no conflicts of interest.

## Supporting information


Table S1

## Data Availability

The data that support the findings of this study are available from the corresponding author upon reasonable request.
