# Supplementary material for: Synergistic mediating effect of edible fungal polysaccharides (Auricularia and Tremellan) and Crataegus flavonoids in hyperlipidemic rats
Source: Food Sci Nutr. 2023 Jun 15;11(8):4812–28. doi: 10.1002/fsn3.3459 (PMC10420763; doi:10.1002/fsn3.3459)
Supplement: Supplementary file 1 — Table S1 [file FSN3-11-4812-s001.doc]

Table S1. Chemical composition of polysaccharides.

| Index | Total sugar(%) | Protein(%) | Uronic acid(%) |
| --- | --- | --- | --- |
| AAP | 69.92 | 6.95 | 17.2 |
| TP | 73.50 | 4.55 | 15.1 |

Table S2 Polyphenols of HFL samples determined by UPLC-MS/MS.

| Compounds | CAS | Formula | Ionization model | | Peak area | |
| --- | --- | --- | --- | --- | --- | --- |
| Alkaloids |  |  |  | |  | |
| Piperidine | 110-89-4 | C5H11N | [M+H]+ | | 2.08E+06 | |
| Putrescine | 110-60-1 | C4H12N2 | | [M+H]+ | | 7.34E+04 |
| L-Azetidine-2-carboxylic acid | 2133-34-8 | C4H7NO2 | | [M+H]+ | | 2.93E+05 |
| Cadaverine | 462-94-2 | C5H14N2 | | [M+H]+ | | 6.32E+04 |
| Choline | 62-49-7 | C5H14NO+ | [M]+ | | | 1.68E+06 |
| Diethanolamine | 111-42-2 | C4H11NO2 | [M+H]+ | | | 3.13E+04 |
| Histamine | 51-45-6 | C5H9N3 | [M+H]+ | | | 2.16E+04 |
| N-Benzylmethylene isomethylamine | - | C8H9N | [M+H]+ | | | 1.84E+06 |
| 2-Phenylethylamine | 156-28-5 | C8H11N | [M+H]+ | | | 1.63E+06 |
| N-Acetylputrescine | 18233-70-0 | C6H14N2O | [M+H]+ | | | 2.66E+04 |
| Agmatine | 306-60-5 | C5H14N4 | [M+H]+ | | | 2.46E+04 |
| N-benzylformamide | 6343-54-0 | C8H9NO | [M+H]+ | | | 1.26E+05 |
| 6-Methylnicotinamide | 6960-22-1 | C7H8N2O | [M+H]+ | | | 6.15E+04 |
| L-Tyramine | 51-67-2 | C8H11NO | [M+H]+ | | | 6.75E+05 |
| 6-Hydroxynicotinic acid | 5006-66-6 | C6H5NO3 | [M+H]+ | | | 1.38E+05 |
| O-Phosphorylethanolamine | 1071-23-4 | C2H8NO4P | [M-H]- | | | 7.32E+04 |
| Histidinol | 501-28-0 | C6H11N3O | [M+H]+ | | | 2.77E+07 |
| N-Acetylcadaverine | 32343-73-0 | C7H16N2O | [M+H]+ | | | 9.01E+05 |
| Indole-3-carboxaldehyde | 487-89-8 | C9H7NO | [M+H]+ | | | 3.24E+04 |
| Dopamine | 51-61-6 | C8H11NO2 | [M+H]+ | | | 1.24E+05 |
| 4,6-Dihydroxyquinoline | 3517-61-1 | C9H7NO2 | [M+H]+ | | | 3.51E+04 |
| Indole-3-carboxylic acid | 771-50-6 | C9H7NO2 | [M-H]- | | | 1.06E+04 |
| DL-2-Aminoadipic acid | 542-32-5 | C6H11NO4 | [M+H]+ | | | 1.17E+05 |
| Indole-3-acetic acid (IAA) | 87-51-4 | C10H9NO2 | [M+H]+ | | | 1.25E+04 |
| 3-Indoleacrylic acid | 1204-06-4 | C11H9NO2 | [M+H]+ | | | 1.73E+06 |
| N-(4-Aminobutyl)benzamide | 5692-23-9 | C11H16N2O | [M+H]+ | | | 7.94E+04 |
| Spermine | 71-44-3 | C10H26N4 | [M+H]+ | | | 2.62E+07 |
| O-Acetyl-L-carnitine | 3040-38-8 | C9H17NO4 | [M+H]+ | | | 2.20E+05 |
| 1-Methoxy-indole-3-acetamide | - | C11H12N2O2 | [M+H]+ | | | 4.40E+06 |
| Methoxyindoleacetic acid | 3471-31-6 | C11H11NO3 | [M+H]+ | | | 6.81E+04 |
| Methyl dioxindole-3-acetate | 57061-18-4 | C11H11NO4 | [M+H]+ | | | 8.54E+04 |
| N-Caffeoylputrescine | 29554-26-5 | C13H18N2O3 | [M+H]+ | | | 2.67E+05 |
| N-Feruloylputrescine | 501-13-3 | C14H20N2O3 | [M+H]+ | | | 9.86E+04 |
| Stearamide | 124-26-5 | C18H37NO | [M+H]+ | | | 1.57E+05 |
| Nicotianamine | 34441-14-0 | C12H21N3O6 | [M+H]+ | | | 1.88E+04 |
| N-Oleoylethanolamine | 111-58-0 | C20H39NO2 | [M+H]+ | | | 7.99E+04 |
| 10-Formyltetrahydrofolic Acid | 2800-34-2 | C20H23N7O7 | [M+H]+ | | | 1.05E+05 |
| N1,N8-Bis(sinapoyl)spermidine | - | C29H39N3O8 | [M+H]+ | | | 3.93E+06 |
| **Flavonoids** |  |  |  | | |  |
| 3',4',7-Trihydroxyflavone | 2150-11-0 | C15H10O5 | [M+H]+ | | | 9.00E+00 |
| Naringenin (5,7,4'-Trihydroxyflavanone) | 480-41-1 | C15H12O5 | [M-H]- | | | 1.92E+04 |
| Phloretin | 60-82-2 | C15H14O5 | [M-H]- | | | 8.18E+05 |
| Epiafzelechin | 24808-04-6 | C15H14O5 | [M+H]+ | | | 2.68E+04 |
| Kaempferol (3,5,7,4'-Tetrahydroxyflavone) | 520-18-3 | C15H10O6 | [M+H]+ | | | 1.08E+04 |
| Luteolin (5,7,3',4'-Tetrahydroxyflavone) | 491-70-3 | C15H10O6 | [M-H]- | | | 2.48E+04 |
| Eriodictyol (5,7,3',4'-Tetrahydroxyflavanone) | 552-58-9 | C15H12O6 | [M-H]- | | | 1.12E+05 |
| Epicatechin | 490-46-0 | C15H14O6 | [M+H]+ | | | 3.30E+06 |
| 4'-Hydroxy-5,7-dimethoxyflavanone | - | C17H16O5 | [M-H]- | | | 1.57E+07 |
| 6-Hydroxyluteolin | 18003-33-3 | C15H10O7 | [M-H]- | | | 1.05E+05 |
| Morin | 480-16-0 | C15H10O7 | [M-H]- | | | 8.27E+06 |
| Quercetin | 117-39-5 | C15H10O7 | [M+H]+ | | | 5.79E+05 |
| Dihydrokaempferide | 137225-59-3 | C16H14O6 | [M+H]+ | | | 2.41E+05 |
| Taxifolin(Dihydroquercetin) | 480-18-2 | C15H12O7 | [M-H]- | | | 1.26E+05 |
| 3'-O-Methyl-epicatechin | 76549-34-3 | C16H16O6 | [M+H]+ | | | 9.00E+00 |
| Epigallocatechin | 970-74-1 | C15H14O7 | [M-H]- | | | 3.79E+04 |
| Gallocatechin | 970-73-0 | C15H14O7 | [M+H]+ | | | 4.68E+05 |
| Dihydromyricetin (Ampelopsin) | 27200-12-0 | C15H12O8 | [M-H]- | | | 2.49E+04 |
| Pinnatifinoside A | - | C21H18O9 | [M+H]+ | | | 5.24E+04 |
| Apigenin-6-C-rhamnoside | - | C21H20O9 | [M+H]+ | | | 3.67E+04 |
| Apigenin-4'-O-glucoside* | - | C21H20O10 | [M+H]+ | | | 1.01E+04 |
| Apigenin-6-C-glucoside (Isovitexin) | 38953-85-4 | C21H20O10 | [M-H]- | | | 3.41E+05 |
| Apigenin-7-O-glucoside(Cosmosiin)* | 578-74-5 | C21H20O10 | [M+H]+ | | | 1.01E+04 |
| Kaempferol-3-O-rhamnoside (Afzelin)(Kaempferin) | 482-39-3 | C21H20O10 | [M-H]- | | | 1.77E+05 |
| Quercetin-3-O-xyloside (Reynoutrin)* | 549-32-6 | C20H18O11 | [M+H]+ | | | 4.91E+06 |
| Morin-3-O-lyxoside* | - | C20H18O11 | [M-H]- | | | 3.45E+06 |
| Avicularin(Quercetin-3-O-α-L-arabinofuranoside)* | 572-30-5 | C20H18O11 | [M+H]+ | | | 4.27E+06 |
| Morin-3-O-arabinoside* | - | C20H18O11 | [M-H]- | | | 3.57E+06 |
| Naringenin-6-C-Glucoside | 3682-03-9 | C21H22O10 | [M+H]+ | | | 9.61E+04 |
| Dihydrocharcone-4'-O-glucoside | - | C21H24O10 | [M-H]- | | | 8.38E+05 |
| Phloretin-4'-O-glucoside (Trilobatin) | 4192-90-9 | C21H24O10 | [M-H]- | | | 8.60E+06 |
| Epicatechin gallate | 1257-08-5 | C22H18O10 | [M+H]+ | | | 1.95E+04 |
| Isorhamnetin-3-O-arabinoside | - | C21H20O11 | [M+H]+ | | | 2.66E+04 |
| Kaempferol-3-O-glucoside (Astragalin)* | 480-10-4 | C21H20O11 | [M+H]+ | | | 8.89E+06 |
| Luteolin-3'-O-glucoside* | 5154-41-6 | C21H20O11 | [M+H]+ | | | 1.04E+07 |
| Kaempferol-4'-O-glucoside* | - | C21H20O11 | [M+H]+ | | | 6.82E+05 |
| Quercetin-3-O-rhamnoside(Quercitrin) | 522-12-3 | C21H20O11 | [M+H]+ | | | 5.45E+05 |
| Luteolin-8-C-glucoside (Orientin) | 28608-75-5 | C21H20O11 | [M+H]+ | | | 1.14E+05 |
| Luteolin-4'-O-glucoside* | 6920-38-3 | C21H20O11 | [M+H]+ | | | 7.40E+06 |
| Luteolin-6-C-glucoside (Isoorientin) | 4261-42-1 | C21H20O11 | [M+H]+ | | | 9.06E+04 |
| Dihydrokaempferol-7-O-glucoside | - | C21H22O11 | [M-H]- | | | 1.84E+05 |
| Eriodictyol-3'-O-glucoside | - | C21H22O11 | [M-H]- | | | 3.89E+05 |
| Dihydrokaempferol-3-O-glucoside | 1049-08-8 | C21H22O11 | [M+H]+ | | | 7.87E+04 |
| Taxifolin-3-O-rhamnoside (Astilbin) | 29838-67-3 | C21H22O11 | [M-H]- | | | 5.35E+04 |
| Cinchonain Ib | 85081-24-9 | C24H20O9 | [M+H]+ | | | 2.22E+06 |
| Cinchonain Id | 85022-67-9 | C24H20O9 | [M-H]- | | | 2.81E+06 |
| Cinchonain Ia | 85022-69-1 | C24H20O9 | [M+H]+ | | | 2.18E+06 |
| Epicatechin glucoside | - | C21H24O11 | [M-H]- | | | 1.98E+06 |
| Epicatechin-3'-O-β-D-glucopyranoside | - | C21H24O11 | [M-H]- | | | 1.35E+06 |
| Epicatechin-3-(3''-O-methyl)gallate | - | C23H20O10 | [M-H]- | | | 6.14E+04 |
| Gallocatechin gallate | 4233-96-9 | C22H18O11 | [M+H]+ | | | 1.62E+04 |
| Epigallocatechin-3-gallate | 989-51-5 | C22H18O11 | [M+H]+ | | | 1.62E+04 |
| Luteolin-7-O-glucuronide | 29741-10-4 | C21H18O12 | [M+H]+ | | | 3.37E+05 |
| Quercetin-3-O-glucoside (Isoquercitrin)* | 482-35-9 | C21H20O12 | [M-H]- | | | 4.87E+06 |
| Rhodiolgin | 94696-39-6 | C21H20O12 | [M+H]+ | | | 1.03E+06 |
| Isohyperoside* | 35589-21-0 | C21H20O12 | [M+H]+ | | | 6.45E+06 |
| Quercetin-5-O-β-D-glucoside | - | C21H20O12 | [M+H]+ | | | 5.61E+06 |
| Quercetin-4'-O-glucoside (Spiraeoside) | 20229-56-5 | C21H20O12 | [M-H]- | | | 3.86E+07 |
| Quercetin-7-O-glucoside* | 491-50-9 | C21H20O12 | [M-H]- | | | 7.72E+06 |
| 6-Hydroxyluteolin 5-glucoside | - | C21H20O12 | [M-H]- | | | 7.72E+06 |
| Quercetin-3-O-galactoside (Hyperin)* | 482-36-0 | C21H20O12 | [M+H]+ | | | 5.81E+06 |
| Taxifolin-3'-O-glucoside | 31106-05-5 | C21H22O12 | [M-H]- | | | 1.05E+05 |
| Galloylisorhamnetin | - | C23H16O11 | [M+H]+ | | | 4.55E+04 |
| Isorhamnetin-3-O-gallate | - | C23H16O11 | [M+H]+ | | | 4.28E+04 |
| Quercetin-4'-O-glucuronide | 201463-36-7 | C21H18O13 | [M-H]- | | | 5.72E+05 |
| Isorhamnetin-7-O-glucoside (Brassicin)* | 6743-96-0 | C22H22O12 | [M+H]+ | | | 1.49E+06 |
| Rhamnetin-3-O-Glucoside | 27875-34-9 | C22H22O12 | [M+H]+ | | | 1.53E+06 |
| Isorhamnetin-3-O-Glucoside* | 5041-82-7 | C22H22O12 | [M+H]+ | | | 1.63E+06 |
| Quercetagetin-7-O-glucoside(Quercetagitrin) | 548-75-4 | C21H20O13 | [M+H]+ | | | 9.16E+04 |
| 3',5',5,7-Tetrahydroxy-4'-methoxyflavanone-3'-O-glucoside | - | C22H24O12 | [M+H]+ | | | 6.63E+04 |
| Kaempferol-3-O-(6''-acetyl)glucoside | - | C23H22O12 | [M-H]- | | | 3.14E+05 |
| Quercetin-3-O-(6''-O-acetyl)glucoside | - | C23H22O13 | [M+H]+ | | | 1.73E+05 |
| Quercetin-3-O-(6''-O-acetyl)galactoside | - | C23H22O13 | [M-H]- | | | 2.18E+06 |
| Kaempferol-3-O-(6''-malonyl)galactoside* | - | C24H22O14 | [M+H]+ | | | 1.57E+06 |
| Kaempferol-3-O-(6''-malonyl)glucoside* | - | C24H22O14 | [M+H]+ | | | 1.59E+06 |
| Quercetin-7-O-(6''-malonyl)glucoside | - | C24H22O15 | [M+H]+ | | | 9.70E+06 |
| Epicatechin-epiafzelechin | - | C30H26O11 | [M-H]- | | | 2.94E+05 |
| Isorhamnetin-3-O-(6''-malonyl)glucoside | - | C25H24O15 | [M+H]+ | | | 1.68E+05 |
| Myricetin-3-O-(6''-malony)glucoside | - | C24H22O16 | [M+H]+ | | | 7.45E+03 |
| Apigenin-7-O-rutinoside (Isorhoifolin) | 552-57-8 | C27H30O14 | [M+H]+ | | | 2.38E+06 |
| Isovitexin-2''-O-rhamnoside | - | C27H30O14 | [M+H]+ | | | 1.99E+05 |
| Vitexin-2''-O-rhamnoside | 64820-99-1 | C27H30O14 | [M+H]+ | | | 4.17E+06 |
| Kaempferol-3,7-O-dirhamnoside (Kaempferitrin) | 482-38-2 | C27H30O14 | [M+H]+ | | | 4.83E+06 |
| Epitheaflavic acid-3-O-Gallate | 34218-97-8 | C28H20O14 | [M-H]- | | | 3.36E+06 |
| Kaempferol-3-O-sambubioside | 27661-51-4 | C26H28O15 | [M+H]+ | | | 9.67E+04 |
| Luteolin-7-O-glucoside-5-O-arabinoside | - | C26H28O15 | [M+H]+ | | | 1.56E+04 |
| Naringenin-7-O-Neohesperidoside(Naringin) | 10236-47-2 | C27H32O14 | [M-H]- | | | 2.86E+04 |
| Naringenin-7-O-Rutinoside(Narirutin) | 14259-46-2 | C27H32O14 | [M+H]+ | | | 5.90E+04 |
| 8,8'-Methylenebiscatechin | 81555-08-0 | C31H28O12 | [M+H]+ | | | 1.45E+06 |
| 7,3'-Di-O-gallyoltricetiflavan | - | C29H22O14 | [M-H]- | | | 5.91E+04 |
| 7,4'-Di-O-galloyltricetiflavan | - | C29H22O14 | [M-H]- | | | 5.34E+04 |
| Kaempferol-3-O-rutinoside(Nicotiflorin) | 17650-84-9 | C27H30O15 | [M+H]+ | | | 2.35E+05 |
| Vitexin-2''-O-galactoside | - | C27H30O15 | [M+H]+ | | | 4.95E+05 |
| Kaempferol-3-O-glucorhamnoside | 40437-72-7 | C27H30O15 | [M+H]+ | | | 2.48E+05 |
| Apigenin-6,8-di-C-glucoside (Vicenin-2) | 23666-13-9 | C27H30O15 | [M+H]+ | | | 3.08E+05 |
| Kaempferol-3-O-glucoside-7-O-rhamnoside | - | C27H30O15 | [M+H]+ | | | 2.73E+05 |
| Vitexin-2''-O-glucoside | 61360-94-9 | C27H30O15 | [M+H]+ | | | 9.55E+04 |
| Luteolin-7-O-neohesperidoside (Lonicerin) | 25694-72-8 | C27H30O15 | [M+H]+ | | | 2.61E+05 |
| Kaempferol-3-O-neohesperidoside | 32602-81-6 | C27H30O15 | [M+H]+ | | | 2.54E+05 |
| Kaempferol-3-O-rhamnosyl(1→2)glucoside | - | C27H30O15 | [M+H]+ | | | 2.82E+04 |
| Luteolin-6-C-glucoside-7-O-rhamnoside | - | C27H30O15 | [M+H]+ | | | 2.94E+04 |
| Isosaponarin(Isovitexin-4'-O-glucoside) | 19416-87-6 | C27H30O15 | [M+H]+ | | | 1.15E+05 |
| Cyanidin-3-O-(6''-O-p-Coumaroyl)glucoside | - | C30H27O13+ | [M]+ | | | 7.01E+04 |
| Quercetin-3-O-sambubioside | 83048-35-5 | C26H28O16 | [M+H]+ | | | 5.09E+04 |
| Kaempferol-3-O-(6''-galloyl)glucoside | 56317-05-6 | C28H24O15 | [M+H]+ | | | 3.31E+04 |
| Kaempferol-3-O-(6''-galloyl)galactoside | - | C28H24O15 | [M+H]+ | | | 1.06E+05 |
| Gallocatechin-(4α→8)-gallocatechin | - | C30H26O14 | [M+H]+ | | | 1.32E+05 |
| Quercetin-3-O-rutinoside (Rutin)* | 153-18-4 | C27H30O16 | [M+H]+ | | | 7.08E+06 |
| Luteolin-7-O-gentiobioside | - | C27H30O16 | [M+H]+ | | | 1.38E+05 |
| Quercetin-3-O-glucoside-7-O-rhamnoside | - | C27H30O16 | [M+H]+ | | | 7.82E+06 |
| Rhodionidin | - | C27H30O16 | [M+H]+ | | | 2.26E+06 |
| Luteolin-6,8-di-C-glucoside | 29428-58-8 | C27H30O16 | [M+H]+ | | | 5.44E+04 |
| Quercetin-3-O-robinobioside | 52525-35-6 | C27H30O16 | [M-H]- | | | 8.26E+06 |
| Quercetin-7-O-rutinoside* | 147714-62-3 | C27H30O16 | [M+H]+ | | | 8.77E+06 |
| Kaempferol-3,7-O-diglucoside | 25615-14-9 | C27H30O16 | [M+H]+ | | | 1.38E+05 |
| Luteolin-7,3'-di-O-glucoside | 52187-80-1 | C27H30O16 | [M+H]+ | | | 2.97E+04 |
| Quercetin-3-O-neohesperidoside | 117611-67-3 | C27H30O16 | [M+H]+ | | | 2.09E+06 |
| Isoluteolin-6,8-di-C-glucoside | - | C27H30O16 | [M+H]+ | | | 1.67E+04 |
| Kaempferol-6,8-di-C-glucoside | - | C27H30O16 | [M+H]+ | | | 1.23E+04 |
| Isorhamnetin-3-O-rutinoside (Narcissin) | 604-80-8 | C28H32O16 | [M+H]+ | | | 1.84E+05 |
| Sexangularetin-3-O-glucoside-7-O-rhamnoside | - | C28H32O16 | [M+H]+ | | | 2.01E+05 |
| Isorhamnetin-3-O-glucoside-7-O-rhamnoside | - | C28H32O16 | [M+H]+ | | | 1.15E+04 |
| Quercetin-3-O-sophoroside (Baimaside) | 18609-17-1 | C27H30O17 | [M+H]+ | | | 2.23E+06 |
| 6-Hydroxykaempferol-3,6-O-Diglucoside | - | C27H30O17 | [M+H]+ | | | 2.20E+06 |
| 6-Hydroxykaempferol-7,6-O-Diglucoside | - | C27H30O17 | [M+H]+ | | | 1.23E+06 |
| Quercetin-3,7-Di-O-glucoside | - | C27H30O17 | [M+H]+ | | | 2.52E+05 |
| Luteolin-7-O-(6''-malonyl)glucoside-5-O-arabinoside | - | C29H30O18 | [M+H]+ | | | 6.48E+03 |
| Kaempferol-3-O-rutinoside-7-O-rhamnoside | - | C33H40O19 | [M+H]+ | | | 6.09E+04 |
| Quercetin-3-O-rutinoside-7-O-rhamnoside | - | C33H40O20 | [M+H]+ | | | 3.27E+04 |
| Quercetin-3-O-(2''-O-Rhamnosyl)rutinoside | 55696-57-6 | C33H40O20 | [M+H]+ | | | 5.18E+06 |
| Quercetin-3-O-sophoroside-7-O-rhamnoside | - | C33H40O21 | [M+H]+ | | | 1.55E+04 |
| Quercetin-7-O-rutinoside-4'-O-glucoside | - | C33H40O21 | [M+H]+ | | | 3.68E+04 |
| Quercetin-3-O-rutinoside-7-O-glucoside | - | C33H40O21 | [M+H]+ | | | 6.07E+04 |
| Cyanidin-3-O-(6''-O-feruloyl)glucoside-5-O-glucoside | - | C37H39O19+ | [M]+ | | | 9.00E+00 |
| Catechin-catechin-catechin | - | C45H38O18 | [M-H]- | | | 1.60E+06 |
| **Phenolic acids** |  |  |  | | |  |
| Benzamide | 55-21-0 | C7H7NO | [M+H]+ | | | 4.88E+05 |
| 4-Hydroxybenzaldehyde | 123-08-0 | C7H6O2 | [M-H]- | | | 2.43E+05 |
| 2-Phenylethanol | 60-12-8 | C8H10O | [M-H2O+H]+ | | | 6.54E+05 |
| 1,3,5-Benzenetriol | 108-73-6 | C6H6O3 | [M+H]+ | | | 2.77E+06 |
| 4-Hydroxyacetophenone | 99-93-4 | C8H8O2 | [M-H]- | | | 2.16E+04 |
| Phenyl acetate | 122-79-2 | C8H8O2 | [M-H]- | | | 2.51E+04 |
| 4-Hydroxybenzoic acid | 99-96-7 | C7H6O3 | [M-H]- | | | 3.68E+05 |
| Salicylic acid | 69-72-7 | C7H6O3 | [M-H]- | | | 2.16E+05 |
| Tyrosol; 4-Hydroxyphenylethanol | 501-94-0 | C8H10O2 | [M-H]- | | | 2.73E+04 |
| 4-Nitrophenol | 100-02-7 | C6H5NO3 | [M+H]+ | | | 8.58E+06 |
| 1-Naphthol* | 90-15-3 | C10H8O | [M-H]- | | | 3.34E+04 |
| 2-Naphthol* | 135-19-3 | C10H8O | [M-H]- | | | 3.64E+04 |
| Phthalic anhydride | 85-44-9 | C8H4O3 | [M+H]+ | | | 9.83E+05 |
| Cinnamic acid | 140-10-3 | C9H8O2 | [M-H]- | | | 6.41E+03 |
| 2-Methoxy-4-ethenylphenol | 7786-61-0 | C9H10O2 | [M+H]+ | | | 1.43E+05 |
| 4'-Hydroxypropiophenone | 70-70-2 | C9H10O2 | [M-H]- | | | 1.51E+04 |
| 4-Allylcatechol | 1126-61-0 | C9H10O2 | [M-H]- | | | 9.08E+03 |
| p-Coumaryl alcohol | 3690-05-9 | C9H10O2 | [M-H]- | | | 4.39E+05 |
| 3-Methoxybenzoic acid | 586-38-9 | C8H8O3 | [M-H]- | | | 1.69E+04 |
| Methyl 4-hydroxybenzoate | 99-76-3 | C8H8O3 | [M-H]- | | | 2.86E+04 |
| Isovanillin | 621-59-0 | C8H8O3 | [M+H]+ | | | 7.91E+04 |
| 2,3-Dihydroxybenzoic Acid* | 303-38-8 | C7H6O4 | [M-H]- | | | 7.35E+06 |
| 3,4-Dihydroxybenzoic acid (Protocatechuic acid)* | 99-50-3 | C7H6O4 | [M-H]- | | | 7.39E+06 |
| 2,5-Dihydroxybenzoic acid; Gentisic Acid* | 490-79-9 | C7H6O4 | [M-H]- | | | 3.72E+06 |
| 3,4-dihydroxy phenylethanol | - | C8H10O3 | [M+H]+ | | | 1.81E+04 |
| 4-MethoxycinnaMaldehyde | 1963-36-6 | C10H10O2 | [M+H]+ | | | 5.31E+04 |
| Caffeic aldehyde | 141632-15-7 | C9H8O3 | [M+H]+ | | | 2.66E+05 |
| p-Coumaric acid | 501-98-4 | C9H8O3 | [M+H]+ | | | 2.33E+05 |
| 3-Hydroxycinnamic Acid | 14755-02-3 | C9H8O3 | [M-H]- | | | 5.47E+05 |
| 2-(Formylamino)benzoic acid | 3342-77-6 | C8H7NO3 | [M-H]- | | | 1.68E+05 |
| 5,7-Dihydroxy-1(3H)-isobenzofuranone | 27979-58-4 | C8H6O4 | [M-H]- | | | 1.69E+06 |
| Terephthalic acid | 100-21-0 | C8H6O4 | [M-H]- | | | 1.69E+06 |
| 3-(4-Hydroxyphenyl)-propionic acid | 501-97-3 | C9H10O3 | [M-H]- | | | 1.34E+04 |
| 2,3-Dimethoxybenzaldehyde | 86-51-1 | C9H10O3 | [M+H]+ | | | 8.28E+03 |
| 2,6-Dimethoxybenzaldehyde | 3392-97-0 | C9H10O3 | [M-H]- | | | 1.34E+04 |
| Ethylparaben | 120-47-8 | C9H10O3 | [M-H]- | | | 1.45E+04 |
| 1-(4-Methoxyphenyl)-1-propanol | 5349-60-0 | C10H14O2 | [M-H]- | | | 7.59E+04 |
| Homogentisic acid | 451-13-8 | C8H8O4 | [M-H]- | | | 7.38E+04 |
| 3,4-Dihydroxybenzeneacetic acid | 102-32-9 | C8H8O4 | [M-H]- | | | 1.00E+05 |
| Protocatechuic Acid Methyl Ester | 2150-43-8 | C8H8O4 | [M-H]- | | | 7.96E+05 |
| Vanillic acid | 121-34-6 | C8H8O4 | [M-H]- | | | 3.21E+04 |
| 2',4',6'-Trihydroxyacetophenone | 480-66-0 | C8H8O4 | [M-H]- | | | 3.92E+05 |
| 2,3,4-Trihydroxybenzoic acid | 610-02-6 | C7H6O5 | [M-H]- | | | 1.57E+04 |
| 2,4,6-Trihydroxybenzoic acid | 83-30-7 | C7H6O5 | [M+H]+ | | | 6.33E+04 |
| Gallic acid | 149-91-7 | C7H6O5 | [M-H]- | | | 1.35E+06 |
| Trans-4-Hydroxycinnamic Acid Methyl Ester | 19367-38-5 | C10H10O3 | [M-H]- | | | 1.65E+04 |
| 4-Methoxycinnamic acid | 830-09-1 | C10H10O3 | [M-H]- | | | 1.83E+04 |
| 5-Acetylsalicylic acid | 13110-96-8 | C9H8O4 | [M+H]+ | | | 2.16E+05 |
| Caffeic acid | 331-39-5 | C9H8O4 | [M-H]- | | | 1.62E+06 |
| Propyl 4-hydroxybenzoate | 94-13-3 | C10H12O3 | [M-H]- | | | 2.45E+04 |
| Methyl 2,4-dihydroxyphenylacetate | 67828-42-6 | C9H10O4 | [M-H]- | | | 2.36E+04 |
| Vanillic acid methyl ester | 3943-74-6 | C9H10O4 | [M-H]- | | | 3.41E+03 |
| Hydroxyphenyllactic acid | 306-23-0 | C9H10O4 | [M-H]- | | | 3.20E+04 |
| 3-O-Methylgallic Acid | 3934-84-7 | C8H8O5 | [M-H]- | | | 2.91E+04 |
| Antiarol; 3,4,5-Trimethoxyphenol | 642-71-7 | C9H12O4 | [M+H]+ | | | 9.48E+04 |
| Dimethyl phthalate | 131-11-3 | C10H10O4 | [M+H]+ | | | 1.09E+05 |
| Methyl caffeate | 3843-74-1 | C10H10O4 | [M-H]- | | | 7.75E+04 |
| Ferulic acid* | 537-98-4 | C10H10O4 | [M-H]- | | | 4.96E+05 |
| Isoferulic Acid* | 25522-33-2 | C10H10O4 | [M-H]- | | | 7.48E+05 |
| Dihydroferulic Acid | 1135-23-5 | C10H12O4 | [M-H]- | | | 1.57E+04 |
| Syringic acid | 530-57-4 | C9H10O5 | [M-H]- | | | 1.01E+05 |
| 4-Hydroxy-3,5-diisopropylbenzaldehyde | 10537-86-7 | C13H18O2 | [M+H]+ | | | 9.00E+00 |
| 2,4-Di-Tert-Butylphenol* | 96-76-4 | C14H22O | [M-H]- | | | 1.49E+05 |
| 2,6-Di-tert-butylphenol* | 128-39-2 | C14H22O | [M-H]- | | | 1.45E+05 |
| Ferulic acid methyl ester | 2309-07-1 | C11H12O4 | [M-H]- | | | 6.93E+03 |
| Sinapinaldehyde | 4206-58-0 | C11H12O4 | [M-H]- | | | 2.92E+04 |
| Methyl Syringate | 884-35-5 | C10H12O5 | [M-H]- | | | 1.05E+04 |
| Eudesmic acid (3,4,5-trimethoxybenzoic acid) | 118-41-2 | C10H12O5 | [M+H]+ | | | 2.69E+04 |
| Ethyl ferulate | 4046-02-0 | C12H14O4 | [M-H]- | | | 8.97E+03 |
| Sinapic acid | 530-59-6 | C11H12O5 | [M-H]- | | | 2.65E+05 |
| 3,4,5-Trimethoxybenzoic acid methyl ester | 1916-07-0 | C11H14O5 | [M+H]+ | | | 9.00E+00 |
| Stilbostemin B | 162411-67-8 | C15H16O2 | [M+H]+ | | | 9.00E+00 |
| Mucic acid Dimethyl Ester | - | C8H14O8 | [M-H]- | | | 9.29E+05 |
| 3,4,5-Trimethoxycinnamic acid | 90-50-6 | C12H14O5 | [M-H]- | | | 5.95E+03 |
| Dibutyl phthalate* | 84-74-2 | C16H22O4 | [M+H]+ | | | 1.60E+07 |
| Diisobutyl phthalate* | 84-69-5 | C16H22O4 | [M+H]+ | | | 1.50E+07 |
| Butyl isobutyl phthalate* | 17851-53-5 | C16H22O4 | [M+H]+ | | | 1.49E+07 |
| Orcinol glucoside | 21082-33-7 | C13H18O7 | [M-H]- | | | 2.80E+05 |
| 3-Hydroxy-5-Methylphenol-1-O-Glucoside | - | C13H18O7 | [M-H]- | | | 3.02E+05 |
| 4-(3,4,5-Trihydroxybenzoxy)benzoic acid | - | C14H10O7 | [M-H]- | | | 8.62E+06 |
| 4-O-Glucosyl-4-hydroxybenzoic acid | - | C13H16O8 | [M-H]- | | | 1.67E+05 |
| Salicylic acid-2-O-glucoside | 10366-91-3 | C13H16O8 | [M-H]- | | | 1.60E+06 |
| Tachioside | 109194-60-7 | C13H18O8 | [M-H]- | | | 1.81E+06 |
| 4-O-Glucosyl-3,4-dihydroxybenzyl alcohol | - | C13H18O8 | [M-H]- | | | 4.76E+05 |
| 1-O-Caffeoyl xylose | - | C14H16O8 | [M-H]- | | | 1.16E+05 |
| 2-Acetyl-3-hydroxyphenyl-1-O-glucoside | - | C15H20O7 | [M-H]- | | | 2.81E+06 |
| Methyl salicylate-2-O-glucoside | 10019-60-0 | C14H18O8 | [M-H]- | | | 5.18E+05 |
| 1-O-Galloyl-rhamnose | - | C13H16O9 | [M+H]+ | | | 9.00E+00 |
| Protocatechuic acid-4-O-glucoside* | - | C13H16O9 | [M-H]- | | | 5.77E+06 |
| 1-O-Gentisoyl-β-D-glucoside* | 23445-11-6 | C13H16O9 | [M-H]- | | | 7.60E+06 |
| Vanilloloside | 74950-96-2 | C14H20O8 | [M-H]- | | | 6.63E+06 |
| 5-(2-Hydroxyethyl)-2-O-glucosylphenol | - | C14H20O8 | [M-H]- | | | 3.06E+06 |
| Cimidahurinine | 142542-89-0 | C14H20O8 | [M-H]- | | | 2.85E+06 |
| 3-O-p-Coumaroylshikimic acid | - | C16H16O7 | [M-H]- | | | 1.61E+05 |
| Digallic Acid | 536-08-3 | C14H10O9 | [M-H]- | | | 1.66E+06 |
| 1-O-p-Coumaroyl-β-D-glucose | 7139-64-2 | C15H18O8 | [M-H]- | | | 5.18E+06 |
| (2E)-3-[4-(β-D-glucopyranoside)-phenylacrylic]-acid | - | C15H18O8 | [M-H]- | | | 5.00E+05 |
| p-Coumaric acid-4-O-glucoside | 117405-48-8 | C15H18O8 | [M-H]- | | | 3.15E+05 |
| Raspberryketone glucoside | 38963-94-9 | C16H22O7 | [M-H]- | | | 3.84E+05 |
| Citrusin C | 18604-50-7 | C16H22O7 | [M-H]- | | | 1.96E+04 |
| isoamericanin A | - | C18H16O6 | [M+H]+ | | | 3.24E+04 |
| Demethyl coniferin | - | C15H20O8 | [M-H]- | | | 4.51E+05 |
| Vanillic acid-4-O-glucoside | 32142-31-7 | C14H18O9 | [M-H]- | | | 9.64E+06 |
| 1-O-Vanilloyl-D-Glucose | - | C14H18O9 | [M-H]- | | | 2.09E+05 |
| Gallic acid-4-O-glucoside | 84274-52-2 | C13H16O10 | [M-H]- | | | 4.29E+05 |
| 6-O-Galloyl-D-glucose* | 13186-19-1 | C13H16O10 | [M-H]- | | | 1.09E+06 |
| 2-O-Galloyl-D-glucose | - | C13H16O10 | [M-H]- | | | 8.68E+05 |
| 3-O-Galloyl-D-glucose* | - | C13H16O10 | [M-H]- | | | 1.15E+06 |
| Koaburaside | 41653-73-0 | C14H20O9 | [M-H]- | | | 1.42E+06 |
| Galloyl Methyl gallate | - | C15H12O9 | [M-H]- | | | 1.96E+05 |
| 5-O-Caffeoylshikimic acid | 180981-12-8 | C16H16O8 | [M-H]- | | | 2.30E+05 |
| 1-O-p-Coumaroylquinic acid | - | C16H18O8 | [M-H]- | | | 7.37E+04 |
| 3-O-p-Coumaroylquinic acid* | 87099-71-6 | C16H18O8 | [M-H]- | | | 7.77E+06 |
| 5-O-p-Coumaroylquinic acid* | 1899-30-5 | C16H18O8 | [M-H]- | | | 3.93E+06 |
| 1-O-Caffeoyl-β-D-glucose | 14364-08-0 | C15H18O9 | [M-H]- | | | 1.12E+06 |
| Syringaldehyde-4-O-glucoside | - | C15H20O9 | [M-H]- | | | 4.08E+05 |
| Dihydrocaffeoylglucose | - | C15H20O9 | [M-H]- | | | 2.69E+05 |
| 1-Caffeoylquinic acid | 1241-87-8 | C16H18O9 | [M-H]- | | | 2.02E+07 |
| Cryptochlorogenic acid (4-O-Caffeoylquinic acid) | 905-99-7 | C16H18O9 | [M-H]- | | | 3.04E+06 |
| Chlorogenic acid (3-O-Caffeoylquinic acid) | 327-97-9 | C16H18O9 | [M-H]- | | | 1.80E+06 |
| Neochlorogenic acid (5-O-Caffeoylquinic acid) | 906-33-2 | C16H18O9 | [M-H]- | | | 2.47E+06 |
| 4-O-β-D-glucopyranosylferulic acid | - | C16H20O9 | [M-H]- | | | 2.39E+06 |
| Homovanilloylquinic acid | - | C16H20O9 | [M-H]- | | | 1.04E+04 |
| 1-O-Feruloyl-β-D-glucose | 7196-71-6 | C16H20O9 | [M-H]- | | | 2.47E+05 |
| Ferulic acid-4-O-glucoside | 117405-51-3 | C16H20O9 | [M-H]- | | | 3.09E+06 |
| Glucosyringic Acid | - | C15H20O10 | [M-H]- | | | 9.04E+05 |
| Chlorogenic acid methyl ester | 29708-87-0 | C17H20O9 | [M-H]- | | | 6.33E+05 |
| 3-Prenyl-4-O-glucosyloxy-4-hydroxybenzoic acid | - | C18H24O8 | [M-H]- | | | 3.10E+05 |
| Quinacyl syringic acid | - | C16H20O10 | [M-H]- | | | 2.97E+04 |
| Trihydroxycinnamoylquinic acid | - | C16H20O10 | [M-H]- | | | 1.14E+05 |
| Awsoniaside B | - | C17H24O9 | [M-H]- | | | 1.19E+05 |
| 4-O-Glucosyl-sinapate | - | C17H22O10 | [M-H]- | | | 1.41E+06 |
| 1-O-Sinapoyl-D-glucose | - | C17H22O10 | [M-H]- | | | 6.79E+05 |
| Bis(2-ethylhexyl)phthalate* | 117-81-7 | C24H38O4 | [M+H]+ | | | 5.49E+07 |
| Diisooctyl Phthalate* | 27554-26-3 | C24H38O4 | [M+H]+ | | | 5.49E+07 |
| Fortuneanoside A | - | C19H22O9 | [M-H]- | | | 7.01E+04 |
| Cimicifugic acid K | - | C20H18O9 | [M-H]- | | | 3.72E+06 |
| Cimicifugic acid C | 205114-67-6 | C20H18O10 | [M-H]- | | | 2.02E+06 |
| Benzyl-β-gentiobioside | 56775-64-5 | C19H28O11 | [M-H]- | | | 3.12E+04 |
| Dunalianoside B | - | C21H22O11 | [M-H]- | | | 2.83E+05 |
| 2-O-Salicyl-6-O-Galloyl-D-Glucose | - | C20H20O12 | [M-H]- | | | 6.96E+04 |
| Flavogallonic Acid Dilactone | 103744-88-3 | C21H10O12 | [M-H]- | | | 1.58E+04 |
| Anthranilate-1-O-Sophoroside | - | C19H27NO12 | [M-H]- | | | 2.12E+05 |
| Trigallic acid | 2131-66-0 | C21H14O13 | [M-H]- | | | 7.79E+05 |
| Monogalloyl-diglucose | - | C19H26O15 | [M-H]- | | | 1.87E+04 |
| 3,6-Di-O-caffeoyl glucose | - | C24H24O12 | [M-H]- | | | 1.56E+04 |
| 1,3-O-Dicaffeoylquinic Acid (Cynarin) | 30964-13-7 | C25H24O12 | [M-H]- | | | 7.78E+05 |
| 6'-O-Feruloyl-D-sucrose | 118230-77-6 | C22H30O14 | [M-H]- | | | 1.13E+04 |
| Rosmarinic acid-3'-O-glucoside | 910028-78-3 | C24H26O13 | [M-H]- | | | 2.24E+05 |
| 3,4-Di-O-caffeoylquinic acid methyl ester | 114637-83-1 | C26H26O12 | [M+H]+ | | | 1.98E+04 |
| 6'-O-Sinapoylsucrose | - | C23H32O15 | [M-H]- | | | 1.61E+04 |
| Disinapoyl glucoside | - | C28H32O14 | [M-H]- | | | 9.00E+00 |
| Tetragallic Acid(3''-O-galloyl-4''-O-galloyl-4-O-galloyl-galloyl acid) | - | C28H18O17 | [M-H]- | | | 1.58E+04 |
| Syringoylcaffeoylquinic acid-D-glucose | - | C31H36O18 | [M-H]- | | | 2.41E+05 |
